# Temperature-dependent photostasis and nitrogen limitation in streamlined-genome red algae Cyanidiophyceae from natural habitats

**DOI:** 10.1093/ismejo/wrag105

**Published:** 2026-04-30

**Authors:** Dai Tsujino, Takayuki Fujiwara, Shota Yamashita, Kei Tamashiro, Jin Izumi, Fumi Yagisawa, Baifeng Zhou, Shunsuke Hirooka, Yuki Sunada, Kintake Sonoike, Shin-ya Miyagishima

**Affiliations:** Department of Gene Function and Phenomics, National Institute of Genetics, 1111 Yata, Mishima, Shizuoka 411-8540, Japan; Genetics Program, Graduate University for Advanced Studies (SOKENDAI), 1111 Yata, Mishima, Shizuoka 411-8540, Japan; Department of Gene Function and Phenomics, National Institute of Genetics, 1111 Yata, Mishima, Shizuoka 411-8540, Japan; Genetics Program, Graduate University for Advanced Studies (SOKENDAI), 1111 Yata, Mishima, Shizuoka 411-8540, Japan; Department of Gene Function and Phenomics, National Institute of Genetics, 1111 Yata, Mishima, Shizuoka 411-8540, Japan; Integrated Technology Center, University of the Ryukyus, 1 Senbaru, Nishihara-cho, Nakagami-gun, Okinawa 903-0213, Japan; Integrated Technology Center, University of the Ryukyus, 1 Senbaru, Nishihara-cho, Nakagami-gun, Okinawa 903-0213, Japan; Research Facility Center, University of the Ryukyus, 1 Senbaru, Nishihara-cho, Nakagami-gun, Okinawa 903-0213 Japan; Department of Gene Function and Phenomics, National Institute of Genetics, 1111 Yata, Mishima, Shizuoka 411-8540, Japan; Department of Gene Function and Phenomics, National Institute of Genetics, 1111 Yata, Mishima, Shizuoka 411-8540, Japan; Department of Gene Function and Phenomics, National Institute of Genetics, 1111 Yata, Mishima, Shizuoka 411-8540, Japan; Genetics Program, Graduate University for Advanced Studies (SOKENDAI), 1111 Yata, Mishima, Shizuoka 411-8540, Japan; Faculty of Education and Integrated Arts and Sciences, Waseda University, 2-2 Wakamatsu-cho, Shinjuku-ku, Tokyo 162-8480, Japan; Department of Gene Function and Phenomics, National Institute of Genetics, 1111 Yata, Mishima, Shizuoka 411-8540, Japan; Genetics Program, Graduate University for Advanced Studies (SOKENDAI), 1111 Yata, Mishima, Shizuoka 411-8540, Japan

**Keywords:** Cyanidiophyceae, *Cyanidiococcus*, *Cyanidioschyzon*, *Galdieria*, photostasis, sulfuric hot spring

## Abstract

Photosynthetic microorganisms must continuously balance light energy absorption with metabolic demand to maintain photostasis under fluctuating environments. Cyanidiophyceae, unicellular red algae from acidic hot springs with highly streamlined genomes (9–18 Mb), nevertheless thrive across a wide temperature range (20°C–56°C), posing the question of how such minimalist eukaryotic cells sustain photostasis in nature. Here, we combined field observations of natural mats in sulfuric hot springs in Japan with laboratory experiments under habitat-mimicking conditions. Spring-water chemistry remained nearly constant year-round, characterized by low nitrogen availability (<30 μM), whereas temperature varied spatially and seasonally. Growth increased with temperature (up to 47°C) and nearly ceased at 20°C–25°C, yet photosynthetic pigment levels and apparatus components remained largely unchanged, indicating sustained light absorption even under conditions of minimal growth. At low temperatures, photosystem efficiency and regulated energy dissipation decreased, whereas nonregulated dissipation and reactive oxygen species (ROS) increased, indicating excess excitation energy was mainly dissipated through nonregulated pathways. Proteomic and transcriptomic analyses showed accumulation of ROS scavengers and chromosome maintenance/repair proteins at low temperature, suggesting that excess reducing power/adenosine triphosphate (ATP), even after partial energy dissipation, was redirected toward stress mitigation rather than growth. At higher temperatures, nitrogen-deficiency responses emerged, reflecting nitrogen limitation relative to elevated demand for rapid growth. Together, these results reveal a temperature-dependent trade-off in Cyanidiophyceae in natural habitats: oxidative stress at low temperature versus nitrogen limitation at high temperature. Overall, our findings highlight a simple yet robust photostasis strategy and provide environmental and omics resources for studies of this model lineage.

## Introduction

In natural environments, microbial growth rates fluctuate with temperature, nutrient availability, and other factors and often remain near zero for extended periods under suboptimal conditions [1]. When growth ceases, biosynthetic demand declines, yet cells must maintain homeostasis through basal energy metabolism [2]. Thus, cells must balance energy acquisition and consumption under changing conditions. For photosynthetic microorganisms, this balance poses a unique challenge. Light reactions, which extract electrons from water and produce nicotinamide adenine dinucleotide phosphate (NADPH) and ATP, are relatively temperature-insensitive, whereas enzymatic processes that consume these molecules, such as CO₂ fixation, slow at low temperature. As a result, energy demand declines while light-driven energy input continues, requiring regulation of energy absorption and dissipation according to cellular capacity [3].

Maintaining the balance between light energy acquisition and consumption, termed photostasis, is essential for preventing over-reduction of the photosynthetic electron transport chain and the resulting production of reactive oxygen species (ROS) that damage cellular components [4–7]. To maintain photostasis under fluctuating environments, photosynthetic organisms employ multiple strategies, including reducing antenna size, dissipating excess energy via nonphotochemical quenching (NPQ; dissipation of excess excitation energy as heat through specialized pigments and proteins) [8], redistributing excitation energy between photosystem (PS) II and PSI (state transition) [9, 10], and adjusting the PSI-to-PSII ratio [11].

Photostasis mechanisms are well characterized in many phototrophs, which possess multilayered regulatory systems. In contrast, Cyanidiophyceae (the genera *Cyanidioschyzon, Cyanidiococcus, Cyanidium*, and *Galdieria*), unicellular red algae inhabiting sulfuric hot springs, have highly streamlined genomes (8.7–17.8 Mb), encoding fewer than 5000 protein-coding genes, representing roughly 30% of those in the green alga *Chlamydomonas reinhardtii* and 60% of those in *Ostreococcus tauri*, which has a compact genome [12–14]. This extensive genome reduction has been suggested to have occurred through two major phases of streamlining: the first in the common ancestor of red algae, associated with adaptation to nutrient-poor marine environments, and the second in the ancestor of Cyanidiophyceae, reflecting adaptation to thermoacidic environments through minimizing the energetic cost of genome maintenance and expression [15, 16]. Consistent with this genome reduction, Cyanidiophyceae lack several key components of photoprotective systems, including PsbS and LHCSR, which are central NPQ factors in land plants and green algae, and orange carotenoid protein (OCP), which mediates NPQ in cyanobacteria [8]. Their photosynthetic antenna systems are also simplified, with reduced diversity of light-harvesting complex I (LHCI) components [17] and loss of phycoerythrin (a red photosynthetic pigment) and several phycobilisome proteins [18]. Despite these simplifications, Cyanidiophyceae inhabit sulfuric hot springs across a wide temperature range (~20°C–56°C) and remain photosynthetically active even at low temperatures [19, 20]. These features suggest that Cyanidiophyceae possess a remarkably streamlined yet effective photostasis system.

Because photostasis is influenced by inorganic nutrient availability (e.g. nitrogen and phosphorus), which constrains assimilation and the associated cellular energy consumption [21–23], its mechanisms are best understood through analyses reflecting natural habitats rather than nutrient-rich artificial media. Here, we combined field analyses of Cyanidiophyceae in sulfuric hot springs, where thin (<1 mm) mats are dominated by this lineage [24, 25], with laboratory experiments using isolated strains grown under habitat-mimicking conditions. We found that whereas water chemistry remained nearly constant, temperature varied and strongly influenced physiology. Growth declined with decreasing temperature and nearly ceased at 20°C–25°C depending on lineage, whereas photosynthetic components remained largely unchanged. Cyanidiophyceae maintained photostasis at low temperature by dissipating excess light energy mainly through nonregulated mechanisms and allocating remaining energy to stress responses such as ROS detoxification rather than reducing light absorption. In contrast, nitrogen limitation responses emerged at higher temperatures due to increased growth demand. These results reveal (i) a simple photostasis mechanism in Cyanidiophyceae and (ii) a temperature-dependent trade-off between oxidative stress at low temperature and nitrogen limitation at higher temperature. Together, they provide insights into photostasis in a photosynthetic eukaryote with an extremely streamlined genome and establish an environmental and physiological framework for Cyanidiophyceae as an emerging model lineage.

## Materials and methods

### Measuring environmental factors

The environmental factors (temperature, pH, and chemical composition of the hot spring water) at hot spring streams in Sai-no-Kawara, Kusatsu Hot Spring, Kusatsu City, Gunma Prefecture, Japan (36°37′26″ N, 138°35′29″ E), were measured in July 2020, February 2021, May 2021, May 2022, October 2022, December 2022, February 2023, and March 2023. The concentrations of NH_4_^+^, NO_2_^−^, NO_3_^−^, and PO_4_^3−^ were measured using a Water Analyzer for Total-N and Total-P (AACSIII, BL TEC K.K.), and the concentrations of Ca, Fe, K, Mg, Mn, P, and Zn were determined using an inductively coupled plasma (ICP) emission spectrometer (ICPE-9000, Shimadzu) as described previously [24]. The water temperature and pH were measured using a pH meter (DDK-Toa Corp.).

### Isolation, identification, and phylogenetic analyses of *Cyanidiococcus yangmingshanensis* and *Galdieria* sp. from Kusatsu Hot Spring

Water and stones bearing *Cyanidiococcus* spp. and *Galdieria* spp. were collected from the hot spring stream in May 2022 (water temperature 45°C–46°C; pH ~2.0) and July 2020 (water temperature 37°C–42°C; pH ~2.0), respectively. To isolate clones of *Cyanidiococcus*, the samples were serially diluted with MA2 medium (an inorganic medium; pH adjusted to 2.0 with sulfuric acid) [25] supplemented with 100 μM Fe^2+^, dispensed into 96-well plates, and incubated at 42°C under illumination (50 μmol photons m^−2^ s^−1^). To isolate a clone of *Galdieria*, the samples were diluted with MA2 medium, streaked onto 0.5% (w/v) gellan gum–solidified MA2 medium with 100 mM glucose, and incubated at 42°C in the dark. A single colony was then isolated. The resulting clonal cultures were used for further analyses. To identify the lineages to which the isolated *Cyanidiococcus* and *Galdieria* clones belong, *rbcL* genes encoded in their chloroplast genomes were amplified by PCR using the primers 5′-TTGTTCCGCGTAACTCCTCAAC-3′ and 5′-TACGTTAGCTGTTGGTGTTTCTACG-3′, and 5′-TGAGTCAGGAGTAATACCATATGCCAA-3′ and 5′-AAATATTTGCAGTTGGTGTTTCTACA-3′, respectively, and then sequenced. Phylogenetic analyses were performed using the maximum likelihood method implemented in RAxML GUI 2.0 [26, 27]. Details are provided in the legends to Supplementary Figs 2 and 3.

### Stock and batch culture conditions

Stock cultures of *Cc. yangmingshanensis*, its haploid strain used for genome assembly, *G. daedala* established as described above, and *Cyanidioschyzon merolae* MS1 (obtained from Dr Peter Lammers, Arizona State University, USA), were maintained photoautotrophically in 20 ml of Allen’s medium (an inorganic medium) [28] supplemented with 100 μM Fe^3+^ (pH 2.0) at 42°C in 25 cm^2^ tissue culture flasks (TPP Techno Plastic Products) under continuous illumination (50 μmol photons m^−2^ s^−1^) without agitation. For comparison of the cellular growth of *Cc. yangmingshanensis* CcyaKS1 in batch cultures under several medium conditions, natural hot spring water sampled from Kusatsu (hereafter referred to as Sp), Sp supplemented with 40 mM (NH_4_)_2_SO_4_ (Sp + NH_4_^+^), and MA2 medium were filter-sterilized using a 0.22 μm pore-size membrane. Cells from stock cultures (OD_750_ = 2.0–3.0) were collected by centrifugation at 3000 × *g* for 5 min, unless otherwise stated, and gently resuspended in each medium (MA2, Sp, and Sp + NH_4_^+^) to an OD_750_ of 1.0. After resuspension into 30 ml of medium in a 25 cm^2^ tissue culture flask, cultures were incubated on a rotary shaker (120 rpm) at 40°C under continuous illumination (100 μmol photons m^−2^ s^−1^).

### Cultivation in dialysis cassettes

For algal cultivation using the dialysis system, algal cells from stock cultures (OD_750_ = 2.0–3.0) were collected by centrifugation and washed twice with respective medium (MA2 or Sp medium), then gently resuspended in respective medium to an OD_750_ of 2.0. Subsequently, 3 ml of the culture was aseptically injected into a dialysis cassette (Slide-A-Lyzer Dialysis Cassette G3, 20 K, Thermo Fisher Scientific) in a laminar-flow hood, which was placed in a beaker containing 1000 ml of each filter-sterilized medium and a magnetic stir bar, covered with sterile film. The cells were cultured on magnetic stirrers in a temperature-gradient incubator (MTI-1000, Tokyo Rikakikai) under continuous illumination (100 μmol photons m^−2^ s^−1^) at 20°C, 25°C, 30°C, and 40°C. The incubator was not maintained under sterile conditions; however, we confirmed that no microbial contamination occurred in the beakers during the experiment.

### Microscopy

Cells (either from clonal cultures or samples from natural algal mats) were observed by differential interference contrast (DIC) microscopy (BX51; Olympus). To visualize nuclei, 100 μl of culture or natural samples in hot spring water was fixed by adding glutaraldehyde to a final concentration of 1.0% and incubating at room temperature for 10 min. The fixed cells were stained with 1 μg/ml 4′,6-diamidino-2-phenylindole (DAPI) and observed with an epifluorescence microscope (BX51; Olympus). Analysis of cell cycle progression in *Cyanidiococcus* spp. was performed by counting the number of nuclei per cell [29]. To visualize mitochondria, 100 μl of culture was harvested by centrifugation and resuspended in MA medium. The cells were stained with 5 μg/ml 3,3′-dihexyloxacarbocyanine iodide (DiOC_6_) and incubated at room temperature for 30 min, then washed with MA medium and observed with an epifluorescence microscope. Transmission electron microscopy using rapid freezing and freeze-substitution procedures was performed as described previously [30].

### Quantification of chlorophyll *a*, phycocyanin, protein, and cellular carbon, nitrogen, and phosphorus contents, and measurement of cell volume and number

Pigment concentrations of samples from natural algal mats or cultured cells were determined as previously described [31]. Briefly, each culture was diluted with fresh medium to an OD_750_ of 1.0. The absorption spectrum of each culture was then measured in a 10 mm pathlength cuvette using a spectrophotometer (UV-2600; Shimadzu) equipped with an integrating sphere (ISR-2600Plus; Shimadzu). Phycocyanin concentration was calculated as [PC, μg/ml] = 138.5 × A_620_–35.49 × A_678_, as previously described [32]. For chlorophyll *a* and carotenoids, pigments were extracted with *N,N*-dimethylformamide, and absorbance of the extract was measured for calculations using the following equations: [Chl *a*, μg/ml] = [12 × (A_664_ − A_750_) – 3.11 × (A_647_ − A_750_)] and [Carotenoids, μg/ml] = [1000 × (A_470_ − A_750_) − 2.86 × (Chl *a*, μg/ml)]/221, respectively, as previously described [33]. To determine cellular phosphorus content, cell pellets were washed with water and then subjected to acid hydrolysis and oxidative digestion, and phosphate was quantified colorimetrically on a per-cell basis, as described previously [34, 35]. To determine cellular carbon and nitrogen contents, water-washed, freeze-dried cell pellets were analyzed using a CHN analyzer (JM11, J-Science). Cell volume was measured using a Coulter Counter (Multisizer 4e, Beckman Coulter). Cell numbers were counted using a hemocytometer. Protein content was determined by the Bradford method using the XL-Bradford reagent (KY-1031, APRO Science) [36].

### Measurement of respiratory oxygen consumption and photosynthetic oxygen evolution

Rates of respiratory oxygen consumption and photosynthetic oxygen generation in cell cultures at each temperature (25°C, 30°C, and 40°C) were measured using an Oxytherm system equipped with an Oxygraph controller (Hansatech Instruments Ltd). For the measurement of photosynthetic activity, cells were irradiated with light at 100 μmol photons m^−2^ s^−1^, the same photon flux density (PFD) used during cultivation, and 2000 μmol photons m^−2^ s^−1^.

### Measurement of photosynthetic activity using pulse-amplitude modulated fluorometry

Chlorophyll fluorescence was measured with a pulse-amplitude modulated (PAM) fluorometer (WATER-PAM-II, WALZ). All measurements were performed inside an incubator set to the respective culture temperatures, unless otherwise indicated, and during the measurements, cultures (1.5 ml of liquid culture with a chlorophyll *a* concentration of ~3 μg/ml) were mixed with a stirrer paddle to prevent sedimentation. The cultures were dark-acclimated for 15 min, and the minimum fluorescence level (*F*o) was determined. The maximum fluorescence level (*F*m) was determined by a saturating pulse and actinic light in the presence of 10 μM 3-(3,4-dichlorophenyl)-1,1-dimethylurea (DCMU).

For light-responsive measurements, red actinic light (peaking at 630 nm) of different PFDs (25, 45, 65, 90, 125, 190, 285, 420, 625, 820, 1150, and 1500 μmol photons m^−2^ s^−1^; determined with a spherical micro-sensor (US-SQS/L, WALZ) was applied stepwise at 1-min intervals and saturating pulses were applied to monitor the maximum fluorescence of light-acclimated cells (*F*m′) and the steady-state fluorescence (*F*). Because the PFD value determined with a spherical micro-sensor (US-SQS/L, WALZ) was three times higher than a flat quantum sensor (MQ-200, Apogee), which was used to measure PFD in natural habitats and to set the culture PFD, PAM data obtained at 285 μmol photons m^−2^ s^−1^ were used to represent the culturing PFD (100 μmol photons m^−2^ s^−1^). *F*o′, qP, *F*v′/*F*m′’, *Y*(II), *Y*(NPQ), and *Y*(NO) were calculated from *F*o, *F*m, *F*, and *F*m′ using the equations shown below [37, 38]. *F*v/*F*m = (*F*m - *F*o)/*F*m, *F*o′ = *F*o/{(*F*v/*F*m) + (*F*o/*F*m′)}, qP = (*F*m′ - *F*)/(*F*m′ - *F*o′), *F*v′/*F*m′ = (*F*m′ - *F*o′)/*F*m′, *Y*(II) = (*F*m′ - *F*)/*F*m′, *Y*(NPQ) = *F/F*m′ - *F/F*m, and *Y*(NO) = *F/F*m.

For the analysis of cells acclimated to high temperature after low-temperature cultivation, *F*v/*F*m of the cells cultured at 25°C under continuous illumination (100 μmol photons m^−2^ s^−1^) was first determined as described above. The culture was then shifted to 40°C under continuous illumination for 10 days, after which *F*v/*F*m was measured again.

To compare the decreasing rate in *F*v/*F*m at 25°C and 40°C under inhibited chloroplast protein synthesis, we first applied lincomycin, a chloroplast-specific inhibitor, to *Cyanidiococcus*; however, it was ineffective: even at 160 μM, a concentration comparable to those typically used in other organisms, growth rate did not differ from the untreated control, suggesting that chloroplast translation was not inhibited. We therefore used chloramphenicol, which inhibits both chloroplast and mitochondrial protein synthesis. Chloramphenicol has been reported to enhance photodamage mainly through its interaction with PSII, probably via superoxide production [39]. Nevertheless, it has also been shown that chloramphenicol allows qualitative interpretation and comparison of relative changes in D1 turnover between conditions [40], thereby allowing comparison between higher- and lower-temperature cultures. For the chloramphenicol experiment, cells cultured at each temperature were divided into two groups: one served as a control, and the other was treated with chloramphenicol at a final concentration of 100 μg/ml, as used in a previous study of *Cz. merolae* 10D [41]. Each culture was transferred to a 6-well plate (~3 ml per well) and incubated under continuous illumination (100 μmol photons m^−2^ s^−1^) with shaking. *F*v/*F*m was measured at 0, 1, 3, 6, 12, and 24 h after treatment.

### Genomic analysis of *Cc. yangmingshanensis* CcyaKS1

For de novo assembly of the nuclear, chloroplast, and mitochondrial genomes, genomic DNA was extracted from haploid *Cc. yangmingshanensis* CcyaKS1 as previously described [31] and HiFi sequencing (Pacific Biosciences) was outsourced to Macrogen Inc. Reads were first mapped to the organellar genome of *Cc. yangmingshanensis* N3110 [14] using Minimap2 ver. 2.24-r1122 [42], and unmapped reads were retained for nuclear genome assembly. The nuclear genome was assembled with Canu ver. 2.0 [43]; contigs were classified by the presence of telomeric repeats, and chromosome-level scaffolds were obtained by manual refinement guided by basic local alignment search tool (BLAST) (local installation, National Center for Biotechnology Information (NCBI) BLAST+ v2.15.0) [44] comparisons and RNA-seq read mapping using HISAT2 v2.2.1 [45] as described in the following section. Separately, reads that mapped to the organellar genomes were assembled to reconstruct the chloroplast and mitochondrial genomes. For the nuclear genome, 4851 gene models were annotated based on homology to *Cz. merolae* 10D and RNA-seq reads. Gene models for the chloroplast (202 genes) and mitochondrial (34 genes) genomes were annotated based on homology to previously published organellar genome annotations [46, 47] using BLAST searches. Genes related to photosynthetic apparatus, chromosome maintenance proteins, and nucleolar proteins were identified based on Kyoto Encyclopedia of Genes and Genomes (KEGG) pathway annotations and functional classifications, supplemented by homology to annotated proteins in *Cz. merolae*.

### RNA extraction and RNA-seq analysis

RNA extraction and RNA-seq analysis were performed with minor modifications to the method previously described [30]. For RNA extraction from algal mats in natural habitats, algae growing on submerged stones were gently scraped off with a toothbrush, immersed in 10 ml of RNAlater solution, and stored at 4°C. The cells were then harvested by centrifugation at 10 000 × *g* for 20 min, resuspended in distilled water, and centrifuged again. For RNA extraction from cultured algal cells, cells from 3 ml of culture were harvested by centrifugation. In cases where RNA levels were compared on a per-cell basis rather than relative to total messenger RNA (mRNA) in *Cc. yangmingshanensis, Cz. merolae* 10D cells were added as a spike-in control at a 1:10 ratio based on cell number. Total RNA was extracted using sodium dodecyl sulfate–elution buffer (SDS-EB) buffer (0.5% [w/v] SDS, 50 mM Tris-HCl, pH 8.0, 5 mM ethylenediaminetetraacetic acid (EDTA)) and PCI (phenol:chloroform:isoamyl alcohol = 25:24:1), with bead beating at 4°C, followed by standard phenol/chloroform purification and isopropanol precipitation. After DNase I treatment, RNA was re-purified using the same method.

RNA-seq libraries were prepared after ribosomal RNA (rRNA) depletion using the NEBNext rRNA Depletion Kit (New England Biolabs) and sequenced as 150-bp paired-end reads on a NovaSeq 6000 system (Illumina) at Novogene Co., Ltd. After trimming with Trimmomatic v0.39 [48], reads were mapped to the nuclear, plastid, and mitochondrial genomes of *Cc. yangmingshanensis* CcyaKS1 and *Cz. merolae* using HISAT2 v2.2.1 [45]. Gene-level read counts were obtained and normalized using DESeq2 [49], applying the default size factor normalization method (median-of-ratios). Two size factors were calculated: one based on reads derived from the *Cz. merolae* spike-in control, which was used only to estimate mRNA abundance on a per-cell basis, and another based on total CcyaKS1 mRNA read counts for transcriptome-wide comparisons. Differentially expressed genes were defined as those with a false discovery rate (FDR) < 0.05 and |log₂ fold change| > 1 (Wald test).

### Mass spectrometry–based proteome analysis

Data-independent acquisition (DIA) proteome analysis was performed as described previously [30]. Briefly, cells from each culture (~3 ml) were harvested by centrifugation and stored at −80°C until use. Proteins were extracted from the frozen cell samples, digested into peptides using Trypsin Platinum (Promega), followed by reduction with 10 μM tris(2-carboxyethyl)phosphine and alkylation with 40 mM 2-chloroacetamide, and then separated and analyzed using an UltiMate 3000 RSLCnano LC system (Thermo Fisher Scientific) coupled to a Q Exactive HF-X mass spectrometer (Thermo Fisher Scientific) at Kazusa Genome Technologies Inc. Peptide identification and quantitative analysis of mass spectrometry (MS) data were performed using DIA-NN v1.8.1 with precursor and protein FDRs <0.001. A protein FASTA database derived from the *Cc. yangmingshanensis* CcyaKS1 genome was used for peptide identification and relative quantification. Cross-run normalization (retention time (RT)-dependent) in DIA-NN was applied. Quantitative data were analyzed in R for differential expression and volcano plot generation.

### Immunoblot analysis

Immunoblot analysis was performed as previously described [31], with slight modifications. Briefly, total protein was extracted from each sample as follows. Three milliliters of growing cells (OD_750_ = 2–3) were centrifuged, and the resulting pellet was resuspended in 200 μl of sample buffer (2% SDS, 62 mM Tris-HCl, pH 6.8, 100 mM dithiothreitol (DTT), 10% glycerol, and 0.01% bromophenol blue) with glass beads (acid-washed 425–600 μm; Sigma-Aldrich in Merck). The samples were vortexed for 10 min at 4°C, then centrifuged, and the supernatant was used for immunoblot analysis. PsbA (D1) was detected using antibodies against the C-terminal region of PsbA (1,15 000; AS05 084A, Agrisera).

### Relative quantification of hydrophilic metabolites performed using gas chromatography–mass spectrometry

Hydrophilic metabolome analysis was performed as previously described [30]. Briefly, cells were harvested by centrifugation, frozen in liquid nitrogen, and dried using a freeze dryer (FDU-1200; EYELA). Hydrophilic metabolites were extracted from 5 mg of dried sample using 80% methanol, purified with MonoSpin C18 spin columns (GL Sciences Inc.), trimethylsilylated, and analyzed using a GCMS-TQ8050 NX system (Shimadzu) with GCMSsolution software (Shimadzu) at Kazusa Genome Technologies Inc. Metabolite identification was performed using an in-house GC–MS spectral database. Targeted analysis was conducted using multiple reaction monitoring (MRM), with identification based on MRM transitions, ion ratios, and retention times. Retention indices were calculated using a C7–C33 alkane standard mixture. Data were processed using GCMSsolution (Shimadzu) and LabSolutions Insight (Shimadzu).

### Quantification of reduced glutathione and oxidized glutathione

The quantification of glutathione (GSH) and oxidized glutathione (GSSG) was performed using GSSG/GSH Quantification Kit (G257, DOJINDO) according to previously described method [50] with slight modification. Briefly, cells were harvested from ~3 ml culture by centrifugation, washed with phosphate-buffered saline (PBS), resuspended in 5% sulfosalicylic acid solution, and ground by vortexing with glass beads. Then, the sample was centrifuged and the supernatants were used for quantification.

### Quantification of ATP, NADH/NAD, NADPH/NADP, and starch levels

Quantification of ATP, NADH/NAD, and NADPH/NADP was performed using the ATP Assay Kit–Luminescence (A550, DOJINDO), NAD/NADH Assay Kit WST (N509, DOJINDO), and NADP/NADPH Assay Kit WST (N510, DOJINDO), respectively, according to the manufacturer’s instructions with slight modifications. Briefly, cells were harvested from ~3 ml of culture by centrifugation and resuspended in 400 μl of extraction buffer (50 mM Tris-HCl, pH 8.0, 5 mM EDTA, 1% SDS). Equal volumes of PCI and glass beads were added, and the mixture was homogenized using a Multi-Beads Shocker MB2000C (Yasui Kikai) for 10 min at 2500 rpm and 4°C. After centrifugation, the aqueous phase was collected, and an equal volume of PCI was added. The sample was vortexed for 10 s, the homogenate was centrifuged again, and the resulting aqueous phase was used for quantification. Starch quantification was performed using the EnzyChrom Starch Assay Kit (E2ST-100, BioAssay Systems) according to the manufacturer’s instructions.

### Quantification of intracellular reactive oxygen species

Intracellular ROS levels were quantified using a photo-oxidation-resistant 2′,7′-dichlorofluorescin diacetate (DCFH-DA)–based assay (ROS Assay Kit R253; DOJINDO). Cells were harvested from ~3 ml of culture by centrifugation and incubated with 500 μl of DCFH-DA solution (10 μM in the loading buffer provided with the kit) according to the manufacturer’s instructions. After staining for 1 h at 37°C, cells were collected by centrifugation to remove excess dye, resuspended in the original culture medium, and returned to dialysis cultivation at the original growth temperature. Following 30 min of cultivation under continuous illumination (100 μmol photons m^−2^ s^−1^), as recommended in the manufacturer’s instructions, fluorescence was measured using a microplate reader (Molecular Devices, Filter MAX F5). Fluorescence values from unstained samples (cells treated with the loading buffer alone) were used as background controls and subtracted from those of stained samples.

## Results

### Seasonal and temperature-dependent dynamics of Cyanidiophyceae and their habitats

**Figure 1:**
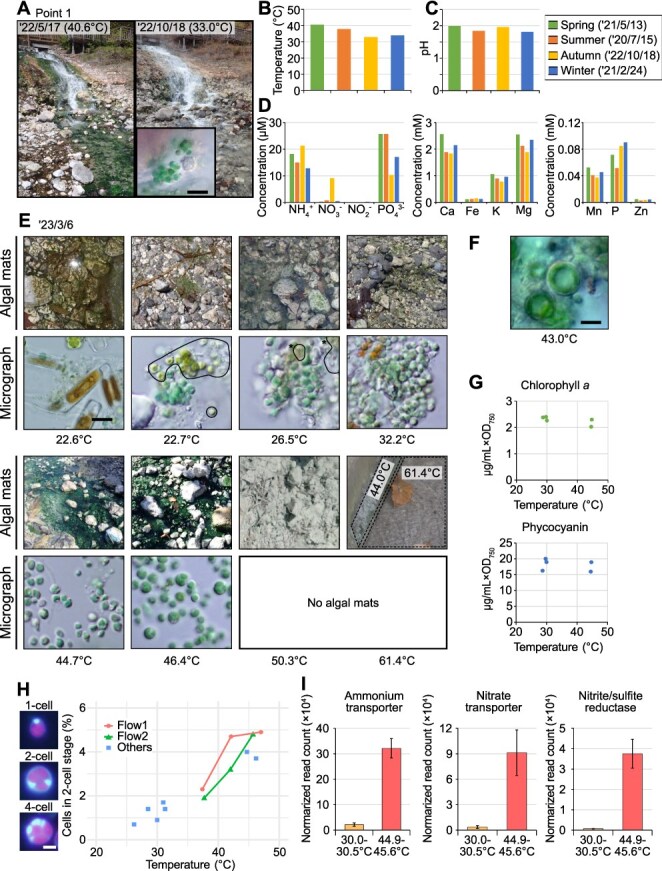
Seasonal changes in the algal mats of cyanidiophycean red algae and the environmental conditions in Sai-no-Kawara, Kusatsu Hot Spring. (A) Landscape view of seasonal changes in cyanidiophycean algal mats at Point 1. The micrograph shows cells in rock crevices on the waterfall wall during periods of reduced mat coverage (Bar = 10 μm). (B–D) Seasonal patterns of environmental factors in hot spring water at Point 1: water temperature (B), pH (C), and chemical composition (D). Data for spring, summer, and winter are identical to those reported previously [24]. Data from other sampling points are shown in Supplementary Fig. 1. (E) Photographs and micrographs of algal mats containing only *Cyanidiococcus* (32.2°C–46.4°C), both *Cyanidiococcus* and other algae (22.7°C and 26.5°C), or only other algae (22.6°C). Bar = 10 μm. Asterisks at 22.7°C and 26.5°C indicate coexisting unicellular green algae. (F) Occasional occurrence of *Galdieria* cells with *Cyanidiococcus*. Bar = 10 μm. (G) Relationship between water temperature and chlorophyll *a* and phycocyanin contents (normalized to OD_750_) of *Cyanidiococcus* mats lacking other algae (26 January 2024; samples collected from 13:30 to 13:50 under cloudy conditions; ~300 μmol m^−2^ s^−1^). (H) Relationship between water temperature and the percentage of *Cyanidiococcus* cells in the two-cell stage (indicator of proliferation rate; *n* ≥ 1000 cells per site). Cells were sampled from six sites in two flows (three points each) from upstream (higher temperature) to downstream (lower temperature) and from seven additional sites in other flows. Representative DAPI-stained images of one-, two-, and four-cell stages are shown left of the graph (chlorophyll fluorescence; DAPI-stained DNA; bar = 2 μm). The percentage of two-cell-stage cells showed diel oscillation, peaking in the evening (Supplementary Fig. 4); evening values are shown. (I) Relative mRNA levels (RNA-seq reads normalized to total *Cc. yangmingshanensis* reads) of genes involved in ammonium and nitrate assimilation in algal mats at two points along the same flow (upstream with higher temperature and downstream with lower temperature). Means ± SD of three time points (10:14, 13:38, and 17:03) are shown. Complete RNA-seq results are summarized in Supplementary Table 2.

To investigate environmental factors affecting the growth and physiology of Cyanidiophyceae in nature, we conducted fixed-site observations at the sulfuric hot spring Sai-no-Kawara in the Kusatsu Hot Spring area, Japan (Supplementary Fig. 1). At locations downstream from the vents where hot water emerges from the ground, such as Point 1 (Fig. 1A), water gradually cools as it flows over the ground and is exposed to air. At these locations, the blue-green algal mat of Cyanidiophyceae (later identified as mainly composed of *Cyanidiococcus yangmingshanensis*; Supplementary Figs 2 and 3) expanded during spring and summer but contracted in autumn and winter (Fig. 1A). Water temperature followed a similar trend, rising above 40°C in the warmer months and dropping below 35°C in the colder ones (Fig. 1B), whereas other environmental parameters, such as pH and inorganic nutrient levels, remained relatively constant throughout the year, with phosphorus showing a comparatively larger fluctuation (Fig. 1C and D; data identical to those previously reported [24], except for autumn; water samples collected from above cyanidiophycean mats). The phosphorus concentrations observed, including PO_4_^3−^ and total P (which includes inorganic forms other than PO_4_^3−^, as well as organic forms), were, even at their lowest levels, well above the reported threshold for hypereutrophic streams (>2.4 μM) [51]. These observations suggest that temperature is a key factor influencing the seasonal growth patterns of *Cyanidiococcus*.

We also examined three other sites (Points 2–4; Supplementary Fig. 1) where mats of Cyanidiophyceae were observed. All sites, including Point 1, received direct sunlight year-round from sunrise to sunset (peaking at ~2000 μmol photons m^−2^ s^−1^) and were shallower than 10 cm. We found that the water composition at these points was also relatively constant throughout the year with only minor spatial differences (Supplementary Fig. 1; water samples collected from above cyanidiophycean mats). Consistent with the general characteristics of sulfuric hot springs [20, 52], the water was reducing with ammonium (NH_4_^+^), rather than nitrate (NO_3_^−^), as the main nitrogen source (Fig. 1D; Supplementary Fig. 1).

To further confirm the positive correlation between temperature and the growth of Cyanidiophyceae mats, we collected algal mats from multiple sites on the same day, covering a range of water temperatures. These sites included three points (upstream, midstream, and downstream) along each of two water flows within a 20 m reach without mixing with other water sources. Thus, water composition within each set of three points was virtually identical, with temperature as the only major variable (higher upstream and lower downstream). As a result, *Cyanidiococcus* was detected at temperatures above 20°C, although it co-occurred with unicellular green algae and diatoms at sites below 27°C (Fig. 1E), as observed in other sulfuric hot springs outside Japan [20, 52, 53]. Occasionally, *Galdieria* coexisted within *Cyanidiococcus* mats (Fig. 1F). No algae, including Cyanidiophyceae, were observed at temperatures above 50°C. At all temperatures at which *Cyanidiococcus* was found (13 sites in total), the cells displayed a similar blue-green color (Fig. 1E). At temperatures above 27°C, the cells contained comparable amounts of chlorophyll *a* and phycocyanin (samples from sites below 27°C, where green algae and diatoms coexisted, were not quantified) (Fig. 1G).

Because direct quantification of cellular growth rates in the field was difficult, we estimated the proliferation rate using the proportion of cells in which a mother cell contained two daughter cells (two-cell stage; Fig. 1H). This approach was based on a previous study showing that once a *Cyanidiococcus* cell reaches a certain threshold size, it undergoes two consecutive divisions without an intervening growth phase [29]. Samples were collected from low-flow areas because algal mats were poorly developed at cooler high-flow sites such as Point 1 in autumn and winter (Fig. 1A). As observed in laboratory cultures under a 12-h light/12-h dark cycle [29], cell division synchronized with the diel cycle was evident at temperatures ≥35°C, where the proportion of two-cell-stage cells peaked in the evening (Supplementary Fig. 4A). The proportion of cells at the two-cell stage (cellular proliferation rate), exhibited a clear positive correlation with water temperature, and this trend was also observed within individual water flows (Fig. 1H). These results show that water temperature is a primary factor influencing cellular growth rate, whereas photosynthetic pigment (chlorophyll *a* and phycocyanin) levels, which reflect light absorption, appear to be less affected by temperature in the natural habitat.

In the natural habitats, the concentrations of inorganic nitrogen sources (Fig. 1D; mainly NH_4_^+^; 10–20 μM) were comparable to those reported for oligotrophic streams (defined as <50 μM [51]). Consistent with this observation, transcriptome (RNA-seq) analysis of cyanidiophycean mats mainly composed of *Cyanidiococcus* showed high expression of nitrogen assimilation genes in fast-growing cells at higher temperatures (44.9°C–45.6°C) during the daytime (mid-morning, noon, and mid-afternoon), when the cells were growing photoautotrophically (Fig. 1I; Supplementary Table 2). These genes, including ammonium transporter, nitrate transporter, and nitrate/sulfite reductase genes, are induced under nitrogen limitation in cyanobacteria and several photosynthetic eukaryotes, including Cyanidiophyceae [54–56]. In Cyanidiophyceae cultures, the nitrate transporter gene is repressed under ammonium-rich conditions (e.g. MA2 medium with 40 mM NH_4_^+^) but strongly induced upon NH_4_^+^ depletion [54, 57]. In contrast, at a downstream site of the same flow, where temperature was lower (30.0°C–30.5°C) and growth was much slower, these genes were only weakly expressed (Fig. 1I; Supplementary Table 2).

### Isolation of *Cyanidiococcus* and *Galdieria* clones and establishment of a cultivation system mimicking the natural habitat

Based on observations in natural habitats, we attempted to reproduce the effects of water temperature on the growth of Cyanidiophyceae under controlled laboratory conditions and to analyze the relationship between temperature and cellular physiology. To this end, cyanidiophycean cells were harvested from Sai-no-Kawara and single clones were isolated. In total, 64 clones were obtained from one sampling point (Point 4 in Supplementary Fig. 1), and two additional clones were isolated from separate points (Supplementary Figs 2 and 3). Phylogenetic analysis based on the *rbcL* sequence identified 65 clones as *Cyanidiococcus yangmingshanensis* (64 from Point 4 and one from an endolithic sample) and one clone as *Galdieria daedala* (GdKS1 from Point 3) (Supplementary Figs 2 and 3). The *Cc. yangmingshanensis* isolates were divided into four clades, with Clade 4 being dominant (56 clones, including CcyaKS1), followed by Clade 2 (8 clones, including CcyaKS4) (Supplementary Fig. 2). Based on these results, CcyaKS1 was selected as a representative strain for subsequent analyses, and CcyaKS4 and GdKS1 were used to examine whether characteristics identified in CcyaKS1 are shared across Cyanidiophyceae.

**Figure 2:**
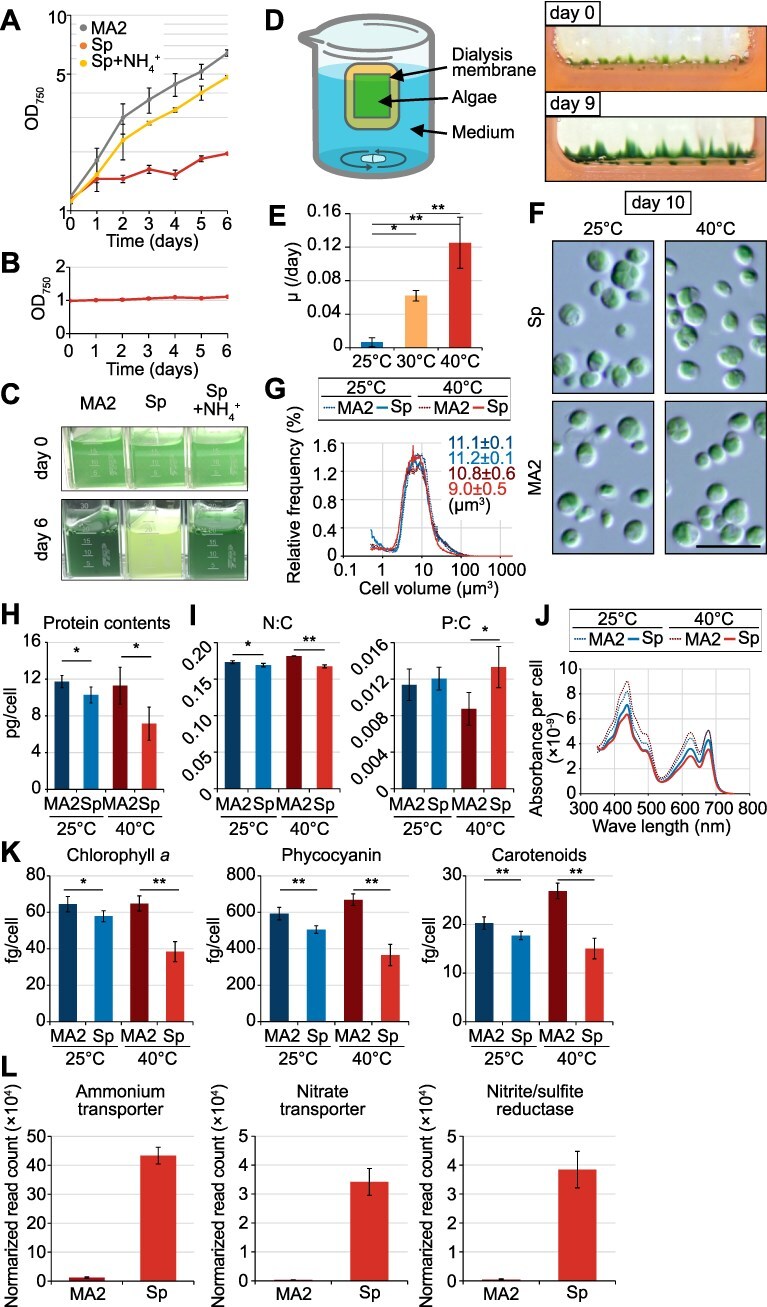
Growth of *Cc. yangmingshanensis* isolated from Sai-no-Kawara in ammonium-rich synthetic inorganic medium and natural hot spring water under batch or dialysis cultivation. (A, B) Changes in OD_750_ in batch cultures of *Cc. yangmingshanensis* CcyaKS1 isolated from sampling point 4 (Supplementary Fig. 1) in MA2 medium (ammonium-rich synthetic inorganic medium), natural hot spring water (Sp medium), and Sp medium supplemented with 80 mM NH_4_^+^ (Sp + NH_4_^+^). Cells from the stock culture were grown for 6 days in each medium (A). Cells previously cultured in Sp medium for 6 days were reinoculated into fresh Sp medium and cultured again (B). Means ± SD of three independent cultures are shown. (C) Photographs of batch cultures in the respective media at 0 and 6 days after inoculation. (D) Illustration of the dialysis cultivation system and photographs of *Cc. yangmingshanensis* CcyaKS1 cultured in Sp medium using this system. (E) Growth rate of CcyaKS1 in dialysis culture in Sp medium at each temperature. Means ± SD of four independent cultures are shown (*P < .05; **P < .01; Tukey’s test). (F) DIC micrographs of *Cc. yangmingshanensis* CcyaKS1 cultured in MA2 and Sp media for 10 days in the dialysis system. Bar = 10 μm. (G–K) Distribution of cell size (measured by Coulter Counter) (G), cellular protein content (H), cellular N:C and P:C ratios (absolute contents are shown in Supplementary Fig. 5A) (I), cellular absorption spectra (normalized to OD_750_) (J), and cellular contents of chlorophyll *a*, phycocyanin, and total carotenoids (K) in CcyaKS1 cultured in MA2 and Sp media for 10 days in dialysis culture. Means ± SD of four independent cultures are shown for H and J (*P < .05; **P < .01; *t-*test). (L) Relative mRNA levels (RNA-seq reads normalized to total *Cc. yangmingshanensis* reads) of genes involved in ammonium and nitrate assimilation in CcyaKS1 cultured in MA2 and Sp media for 10 days in dialysis culture. Means ± SD of four independent cultures are shown. Complete RNA-seq results and DEGs between MA2 and Sp media are summarized in Supplementary Tables 3 and 4.

To establish cultivation conditions mimicking the natural habitat, algae were cultured under batch conditions using natural hot spring water from Sai-no-Kawara (Sp medium). Under batch cultivation at 40°C, the optimal temperature for Cyanidiophyceae in nutrient-rich synthetic media [14, 20], and 100 μmol photons m^−2^ s^−1^, CcyaKS1 grew in nutrient-rich synthetic MA2 medium but not in Sp medium (Fig. 2A–C). After inoculation, cells exhibited limited initial growth (approximately two-fold) in Sp medium, became pale green (Fig. 2A and C), and failed to grow upon transfer to fresh Sp medium (Fig. 2B). In contrast, supplementation of Sp medium with 40 mM ammonium sulfate (equivalent to the NH_4_^+^ concentration in MA2 medium) restored growth to levels comparable to MA2 (Fig. 2A and C), indicating that the low inorganic nitrogen concentration in Sp medium (5–30 μM) is insufficient to sustain growth under batch conditions.

Because fresh hot spring water is continuously supplied to algal mats in nature (Fig. 1A; Supplementary Fig. 1), we next employed a dialysis culture system, in which cells were inoculated into a dialysis cassette submerged in stirred Sp medium to continuously supply the nitrogen-limited medium to the cells (Fig. 2D). Under these conditions, CcyaKS1 grew successfully at 40°C, and similar growth was observed for CcyaKS4 (Fig. 2D and E; Supplementary Fig. 4B).

When CcyaKS1 and CcyaKS4 were cultured in dialysis cassettes at 40°C, 30°C, and 25°C (within the habitat range described above), growth rates, which were estimated from the proportion of two-cell-stage cells, stabilized after Day 7 and remained constant for at least two additional weeks (Supplementary Fig. 4B). Both strains exhibited similar temperature-dependent growth patterns (Fig. 2E; Supplementary Fig. 4B), consistent with field observations (Fig. 1H). Comparable temperature dependence was also observed in dialysis cultures using synthetic MA2 medium, with growth rates nearly identical to those in Sp medium (Fig. 2E).

Despite similar growth rates, CcyaKS1 cultures in Sp medium differed from those in MA2 medium in several respects. Although cell morphology and size were similar (Fig. 2F and G), cellular protein content, nitrogen-to-carbon (N:C) ratio, and chlorophyll *a,* phycocyanin, and carotenoid levels were lower in Sp medium (Fig. 2H–K), which are typical symptoms of nitrogen deficiency in algae including the cyanidiophycean alga *Cz. merolae* [58]. This reduction was more pronounced at 40°C than at 25°C (Fig. 2H–K). In terms of absolute carbon and nitrogen contents per cell, both were reduced by ~50% in Sp medium relative to MA2 medium at 40°C but not at 25°C, and cellular phosphorus content was also ~25% lower at 40°C (Supplementary Fig. 5A). Under simulated midday conditions (100–1500 μmol photons m^−2^ s^−1^ for 4 h), cellular chlorophyll *a*, phycocyanin, and carotenoid levels were also lower in Sp medium, while chlorophyll *a* and carotenoids were reduced overall by 20%–35% relative to 100 μmol photons m^−2^ s^−1^ (Supplementary Fig. 5B). RNA-seq analysis comparing the cells cultured in Sp and MA2 media at 40°C revealed 72 upregulated and 81 downregulated genes in Sp medium relative to MA2 (FDR < 0.05; |log₂ fold change| > 1) (Supplementary Tables 3 and 4). Upregulated genes included those involved in nitrogen assimilation, which are induced under nitrogen limitation, such as ammonium and nitrate transporters and nitrite/sulfite reductase, as well as nitrate reductase and an MYB-related transcription factor known to regulate nitrogen-assimilation genes in *Cz. merolae [**54**]* (Fig. 2L; Supplementary Fig. 5C and D).

### Components of the photosynthetic apparatus, including photosynthetic proteins and pigments, are maintained despite growth inhibition at lower temperatures in *Cyanidiococcus*

The analyses below of temperature-dependent differences in cellular physiology are based on comparisons of *Cc. yangmingshanensis* CcyaKS1 cells grown in Sp medium by dialysis culture for ≥10 days after inoculation at each temperature, when growth had reached a steady state (Fig. 2E).

**Figure 3:**
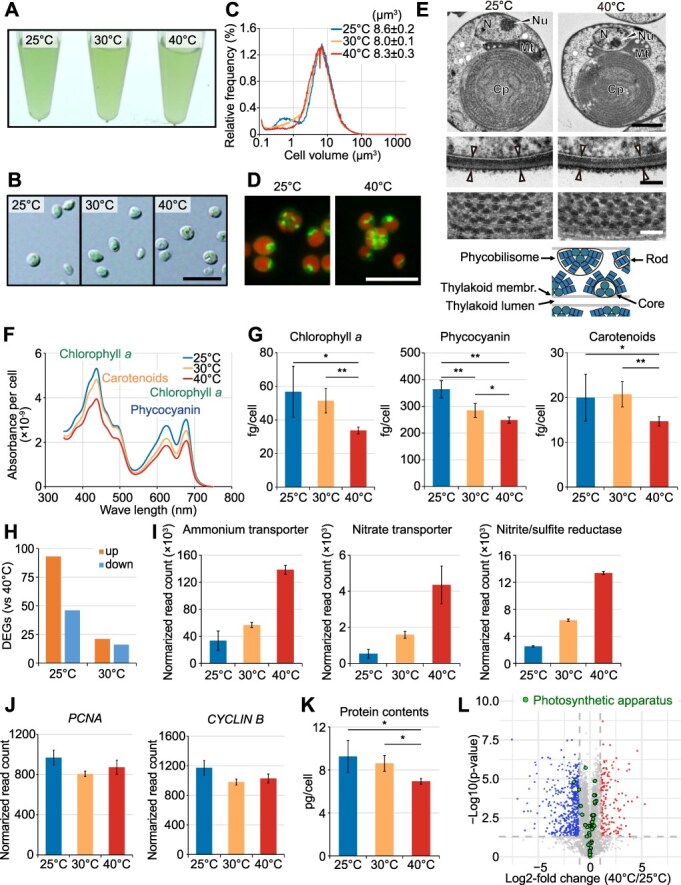
Comparison of *Cc. yangmingshanensis* CcyaKS1 cells cultured at different temperatures in Sp medium for 10 days using the dialysis cultivation system. (A, B) Photographs and DIC micrographs of *the culture* at 40°C, 30°C, and 25°C. Bar = 10 μm. (C) Distribution of cell size (determined by Coulter Counter) at 40°C, 30°C, and 25°C. (D) Mitochondrial and chloroplast morphology of cells at 40°C and 25°C, observed by fluorescence microscopy. Merged images show mitochondria stained with DiOC_6_ and chloroplasts visualized by chlorophyll fluorescence. (E) Transmission electron micrographs of cells at 40°C and 25°C. The middle and lower rows show magnified views focusing on the cell wall (middle) and on the thylakoid membrane arrays and phycobilisomes (lower) of the corresponding images above. *N*, nucleus; *Nu*, nucleolus; *Mt*, mitochondrion; *Cp*, chloroplast. Scale bars = 1 μm (upper), 100 nm (middle and lower). (F) Cellular absorption spectra at different temperatures at 40°C, 30°C, and 25°C. (G) Cellular chlorophyll *a*, phycocyanin, and total carotenoid contents at 40°C, 30°C, and 25°C. Means ± SDs of three independent cultures are shown (*P < .05; **P < .01; Tukey’s test). (H) Number of DEGs (RNA-seq data normalized to total *Cc. yangmingshanensis* reads; FDR < 0.05 and |log₂ fold change| > 1) in cells at 25°C or 30°C compared with 40°C. (I, J) Relative mRNA levels (RNA-seq data normalized to total *Cc. yangmingshanensis* reads) of genes involved in ammonium and nitrate assimilation (I) and cell cycle progression (*PCNA* for S phase and *CYCLIN B* for M phase) (J) at 40°C, 30°C, and 25°C. Means ± SDs of four independent cultures are shown. The complete RNA-seq results and lists of DEGs are summarized in Supplementary Tables 5, 6, and 7, respectively. (K) Cellular protein contents at 40°C, 30°C, and 25°C. Means ± SDs of four independent cultures are shown (*P < .05; **P < .01; Tukey’s test). (L) Volcano plot of the DIA proteome comparing cells at 40°C and 25°C. Red and blue dots represent proteins with higher abundance *P* < .01 and |log₂ fold change| > 1; three independent cultures per condition) at 40°C and 25°C, respectively. Green dots indicate components of the photosynthetic apparatus. The full proteome results and lists of differentially expressed proteins are summarized in Supplementary Tables 8 and 9.

Optical microscopy revealed no obvious morphological differences at any temperature (Fig. 3A and B), consistent with those observed in the natural habitat (Fig. 1). Cell volume remained nearly constant (Fig. 3C), and fluorescence microscopy showed no notable differences in chloroplast or mitochondrial size or shape between 40°C and 25°C (Fig. 3D; >1000 cells analyzed for each condition). Transmission electron microscopy likewise revealed no major differences in intracellular structures (>100 cells analyzed for each condition), except for a larger nucleosome at 25°C (Fig. 3E). Cell wall thickness was similar at both temperatures (~35 nm), as were the number and spacing of thylakoid layers and the size (~24 nm in diameter) and density of phycobilisomes (light-harvesting antenna complexes that mainly transfer excitation energy to PSII) (Fig. 3E). Cellular absorbance spectra were nearly identical across temperatures (Fig. 3F). Cellular chlorophyll *a*, phycocyanin, and total carotenoid levels were lower at 40°C than at 30°C and 25°C (Fig. 3G). Thus, although cells hardly grew at 25°C (Fig. 2E), they were capable of absorbing light comparable to, or greater than, that of well-growing cells at 40°C.

RNA-seq analyses showed that total cellular mRNA levels (nuclear and organellar), normalized using spike-in controls proportional to cell number, were higher at lower temperatures, being approximately three-fold higher at 25°C than at 40°C (Supplementary Fig. 6; Supplementary Tables 5–7). However, when normalized to total *Cc. yangmingshanensis* mRNA reads, overall expression profiles differed little between temperatures. Of 5087 genes, only 93 (1.8%) and 46 (0.9%) were up- and downregulated, respectively, at 25°C relative to 40°C (Fig. 3H). In light of this observation, all subsequent gene expression comparisons were based on values normalized to total mRNA reads rather than per-cell values. Nitrogen assimilation genes constituted the most prominent functional group among the differentially expressed genes (DEGs) (Supplementary Table 7). For genes encoding an ammonium transporter, nitrate transporter, and nitrite/sulfite reductase, mRNA levels were highest at 40°C, lowest at 25°C, and intermediate at 30°C (Fig. 3I). Consistent with this result, proteomic analysis showed that ammonium transporter (CcyaKS1_nu_g3826) and nitrite/sulfite reductase (CcyaKS1_nu_g4340) proteins were ~6- and 40-fold more abundant, respectively, at 40°C than at 25°C (Supplementary Tables 8 and 9). Together with the reduction in cellular photosynthetic pigment levels at 40°C (Fig. 3G), these patterns are consistent with nitrogen supply limitation in Sp medium under increased nitrogen demand at higher temperatures associated with faster growth.

mRNA levels of the cell cycle markers *PCNA* and *CYCLIN B*, which accumulate in S and M phases, respectively [59], remained nearly constant across temperatures (Fig. 3J). This pattern suggests that at low temperature, where proliferation is minimal, cell cycle progression is not arrested at a specific phase (e.g. G1 or G2) but rather slowed across phases.

Cellular protein content was ~27% lower at 40°C than at 30°C and 25°C (Fig. 3K), again consistent with relative nitrogen limitation at higher temperature. DIA proteomics identified 4138 proteins (81.3% of the predicted proteome), of which 425 (10.3%) and 143 (3.5%) were up- and downregulated, respectively, at 25°C compared with 40°C (*P* < .05, |log₂ fold change| > 1) (Fig. 3L; Supplementary Tables 8 and 9), despite minimal transcriptomic differences (Fig. 3H). Nevertheless, the relative abundance of photosynthetic apparatus proteins, including components of PSII, the cytochrome b6f complex, PSI, the phycobilisome, and light-harvesting complex I (LHCI; a PSI-associated antenna complex in red algae) (46 proteins in total based on the KEGG database; pathway IDs cme00195 and cme00196), did not differ significantly between 25°C and 40°C (Fig. 3L; Supplementary Tables 8 and 9; differences in other protein groups are described in Supplementary Results 1).

### Suppression of photosynthetic and respiratory activities at lower temperature in *Cyanidiococcus*

The above results indicate that *Cc. yangmingshanensis* CcyaKS1 cells at 25°C maintained comparable amounts of photosynthetic apparatus components and slightly higher pigment levels, absorbing light similar to or greater than actively growing cells at 40°C. However, because cells hardly grow at low temperatures, their overall energy demand is lower. To examine how cells cope with this imbalance, we compared photosynthetic and respiratory activities at different temperatures.

**Figure 4:**
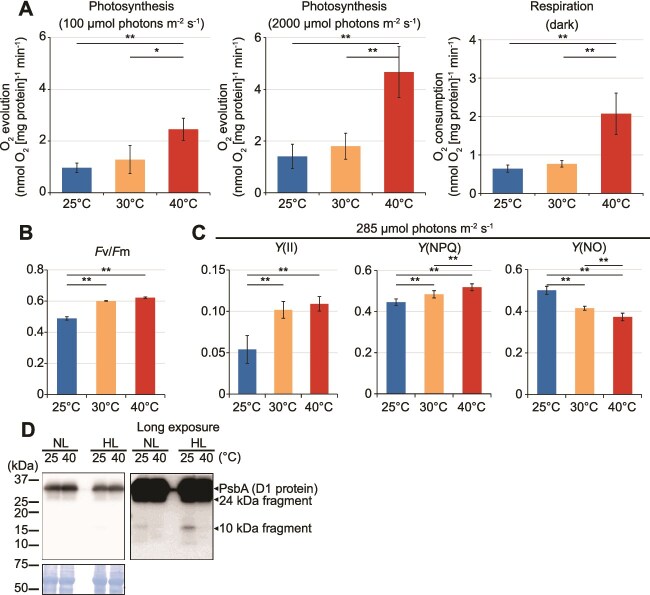
Photosynthetic and respiratory activities and D1 protein profile in *Cc. yangmingshanensis* CcyaKS1 cultured at different temperatures in Sp medium for 10 days using the dialysis cultivation system. (A) Net photosynthetic oxygen evolution at 100 μmol photons m^−2^ s^−1^ (same as the cultivation condition) and 2000 μmol photons m^−2^ s^−1^, and respiratory oxygen consumption in the dark at 40°C, 30°C, and 25°C. Net photosynthetic oxygen evolution was calculated as gross oxygen evolution + |respiratory oxygen consumption in the dark|. (B) Maximum quantum yield of PSII (*F*v/*F*m) in dark-acclimated cells. (C) Effective quantum yield [*Y*(II)], nonphotochemical quenching [*Y*(NPQ)], and nonregulated energy dissipation [*Y*(NO)] measured in light-acclimated cells under actinic light of 285 μmol photons m^−2^ s^−1^, approximately equivalent to the cultivation PFD (see Materials and Methods), at 40°C, 30°C, and 25°C. Means ± SDs of four independent cultures are shown for panels A–C (*P < .05; **P < .01; Tukey’s test). (D) Immunoblot detection of the D1 protein of PSII in cells at 40°C and 25°C under 100 μmol photons m^−2^ s^−1^, and after a shift to 1500 μmol photons m^−2^ s^−1^ for 4 h. Results from both normal and longer exposure times are shown. The same amount (10 μg) of total cellular protein was separated in each lane. A Coomassie Brilliant Blue–stained PVDF membrane is shown as a loading control.

Photosynthetic activity was strongly suppressed at low temperatures. Oxygen evolution (water splitting at PSII, the initial step of photosynthetic electron transport) at 25°C was ~40% of that at 40°C under both the culturing light (100 μmol photons m^−2^ s^−1^) and high light (2000 μmol photons m^−2^ s^−1^), comparable to peak natural irradiance (Fig. 4A; Supplementary Fig. 7A). Dark respiration was similarly reduced to ~35% of the 40°C rate (Fig. 4A; Supplementary Fig. 7A). Thus, oxygen evolution declined at lower temperature despite unchanged levels of photosynthetic pigments and photosynthetic apparatus proteins. To assess whether this reflected altered photochemical performance, we analyzed photosynthetic function using PAM fluorometry. Red excitation (~590–610 nm) was used to excite both chlorophyll *a* and phycobilisomes, reflecting the natural excitation pathway in Cyanidiophyceae. *F*v/*F*m declined with decreasing temperature to ~80% of the 40°C value at 25°C (Fig. 4B; Supplementary Fig. 7B). PSII electron transport [*Y*(II)] (under irradiance close to the culturing light, 285 μmol photons m^−2^ s^−1^; see Materials and Methods) similarly declined to ~50% at 25°C relative to 40°C, consistent with reduced oxygen evolution (Fig. 4C; Supplementary Fig. 7B). Energy partitioning analysis indicated slightly lower NPQ but higher nonregulated dissipation [*Y* (NO)] at low temperature (Fig. 4C; Supplementary Fig. 7B).

The reduction in *F*v/*F*m suggested impaired PSII photochemical efficiency. Given the high susceptibility of the PSII D1 protein (a core reaction-center protein of PSII involved in primary charge separation and water oxidation) to photodamage and its continuous replacement by newly synthesized protein, even under optimal growth conditions [4], we next examined the state of the D1 protein. When damaged PSII centers remain unrepaired, PSII photochemical efficiency declines, reaction centers become closed, and excitation pressure increases. Under such conditions, overexcitation energy within PSII promotes the formation of ROS, mainly singlet oxygen (^1^O₂) generated by triplet chlorophyll in the reaction center [60]. Immunoblotting showed that, in agreement with the proteomic results, total D1 levels were similar between temperatures (Fig. 4D). In contrast, a 10 kDa D1 degradation fragment accumulated at 25°C, to a lesser extent under growth light (100 μmol photons m^−2^ s^−1^) and to a greater extent under high light (1500 μmol photons m^−2^ s^−1^ for 4 h) but not at 40°C (Fig. 4D). This fragment is generated by Deg-mediated cleavage of photodamaged D1 and is subsequently degraded by the FtsH protease [61], indicating a higher proportion of nonfunctional PSII centers at low temperature.

To determine whether the reduced PSII efficiency at low temperature resulted from altered D1 turnover, we monitored *F*v/*F*m after chloramphenicol treatment, which inhibits organellar protein synthesis (lincomycin, a chloroplast-specific inhibitor, was ineffective in *Cyanidiococcus* as described in the Materials and Methods). Decreasing rates of *F*v/*F*m were nearly identical at 25°C and 40°C (Supplementary Fig. 7C), consistent with the idea that PSII photodamage depends primarily on light intensity rather than temperature [4]. In contrast, without chloramphenicol, *F*v/*F*m remained constant at both temperatures (Supplementary Fig. 7C), indicating balanced D1 damage and repair. These results suggest that the reduction in *F*v/*F*m and the higher proportion of nonfunctional PSII centers at low temperature are not due to a general slowing of the entire D1 turnover cycle but instead reflect limitations in one or more specific steps, likely including the proteolytic removal of damaged D1, that are slowed at low temperature.

When cells cultured at 25°C with reduced *F*v/*F*m were shifted to 40°C and further cultured for 10 days, *F*v/*F*m recovered to ~0.6, similar to cells continuously grown at 40°C (Supplementary Fig. 7D), showing that PSII activity reduced at low temperature can be fully restored upon a shift to higher temperature.

### Greater reactive oxygen species and antioxidant accumulation at lower temperature in *Cyanidiococcus*

Nonregulated dissipation [*Y*(NO)] represents the fraction of absorbed light energy dissipated through noncontrolled pathways, including both nonROS-generating and ROS-generating processes [5, 38]. At low temperature, increased *Y*(NO), together with decreased *F*v/*F*m and accumulation of the ~10 kDa D1 fragment, suggests enhanced ROS generation relative to 40°C.

**Figure 5:**
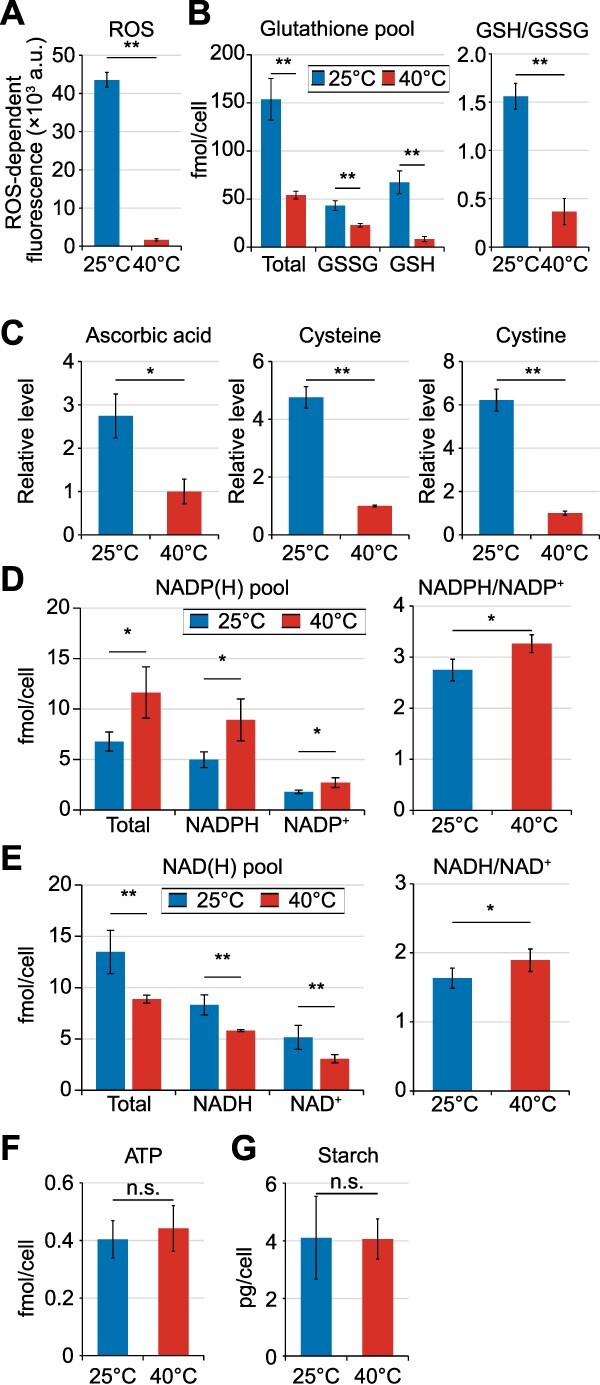
Cellular levels of ROS, antioxidants, reducing equivalents, ATP, and starch in *Cc. yangmingshanensis* CcyaKS1 cultured at 40°C and 25°C in Sp medium for 10 days using the dialysis cultivation system. (A) Cellular ROS levels quantified using photo-oxidation–resistant DCFH-DA. (B) Cellular concentrations of oxidized glutathione (GSSG), reduced glutathione (GSH), and the GSH/GSSG ratio. (C) Relative levels (per dry cell weight) of ascorbic acid, cysteine, and cystine determined by metabolomic analysis. Values at 40°C were set to 1.0. (D) Cellular concentrations of NADPH and NADP^+^, and the NADPH/NADP^+^ ratio. (E) Cellular concentrations of NADH and NAD^+^, and the NADH/NAD^+^ ratio. (F) Cellular ATP concentration. (G) Cellular starch content. Means ± SDs of four independent cultures are shown (*P < .05; **P < .01; *t-*test).

To assess photosynthetic oxidative stress and cellular responses, we compared ROS and antioxidant levels at different temperatures. Total cellular ROS, quantified using a photo-oxidation-resistant DCFH-DA probe, were higher at 25°C than at 40°C (Fig. 5A). Glutathione analysis revealed a larger total pool of reduced (GSH) and oxidized (GSSG) forms and a higher GSH/GSSG ratio at 25°C (Fig. 5B). Metabolomic profiling similarly showed higher levels of antioxidants at 25°C, including ascorbic acid (approximately three-fold), cysteine (approximately five-fold), and cystine (approximately six-fold; oxidized cysteine) (Fig. 5C; Supplementary Fig. 8; Supplementary Table 10). These results indicate enhanced ROS production at low temperature and compensatory accumulation of diverse ROS-scavenging metabolites. In contrast, levels of enzymatic ROS-scavenging proteins such as superoxide dismutase, catalase, and peroxiredoxin did not differ markedly between temperatures (Supplementary Tables 8 and 9; rows corresponding to these proteins are shaded in gray).

### Temperature-dependent reallocation of NAD(H)/NADP(H) pools while maintaining overall energy/redox balance in *Cyanidiococcus*

To compare intracellular energy metabolism and redox state between low and high temperatures, we quantified cellular ATP, NAD(H), and NADP(H) as energy/redox equivalents, and starch as a major energy reserve.

The NADPH/NADP^+^ and NADH/NAD^+^ ratios, ATP levels, and starch contents were nearly identical at 40°C and 25°C (Fig. 5D–G), indicating that intracellular energy and redox balance are maintained despite differences in temperature and growth rate. However, total NADP(H) and NAD(H) pool sizes were lower and higher, respectively, at 25°C than at 40°C (Fig. 5D and E).

### Temperature-dependent responses in other cyanidiophycean lineages similar to those in *Cyanidiococcus*

**Figure 6:**
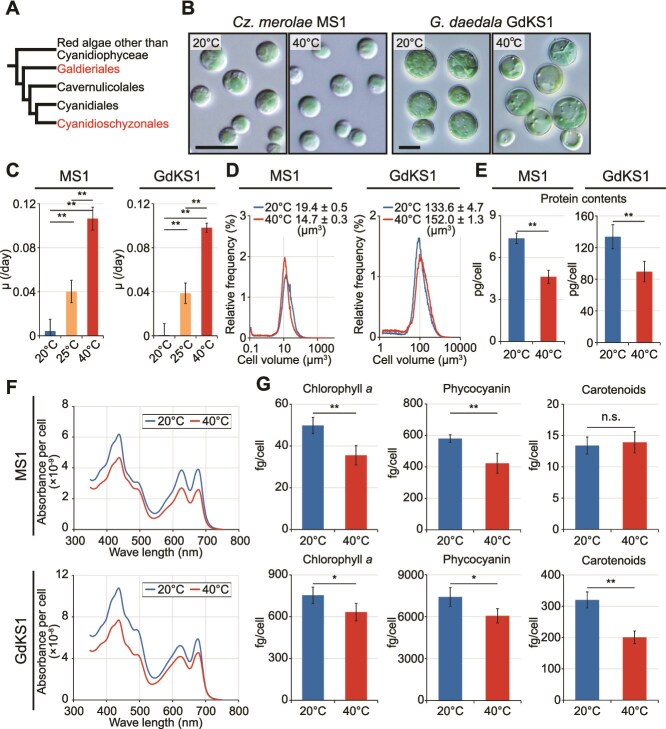
Cellular morphology, growth rates, and photosynthetic pigment levels of *G. daedala* GdKS1 and *Cz. merolae* MS1 cultured at different temperatures in Sp medium for 10 days using a dialysis cultivation system. (A) The phylogenetic relationships of Cyanidiophyceae comprise four orders: Cyanidioschyzonales (including the genera *Cyanidiococcus* and *Cyanidioschyzon*), Cyanidiales, Galdieriales (genus *Galdieria*), and Cavernuliconales. The first three inhabit sulfuric hot springs, whereas Cavernuliconales are secondarily adapted to mesophilic neutral environments and lack cultured strains [46]. (B) DIC micrographs of *G. daedala* GdKS1 and *Cz. merolae* MS1 at 40°C and 20°C. Bars = 10 μm. (C) Growth rates of *G. daedala* GdKS1 and *Cz. merolae* MS1 at 40°C, 25°C, and 20°C. Means ± SDs of four independent cultures are shown (*P < .05; ** *P* < .01; Tukey’s test). (D–G) Distribution of cell size (determined by Coulter Counter) (D), cellular protein content (E), cellular absorption spectra (F), and cellular contents of chlorophyll *a*, phycocyanin, and total carotenoids (G) in *G. daedala* GdKS1 and *Cz. merolae* MS1 at 40°C and 20°C. Means ± SDs of four independent cultures are shown for C, E, and G (*P < .05; ** *P* < .01; *t-*test).

To examine whether temperature-dependent responses observed in *Cyanidiococcus* are conserved across Cyanidiophyceae (Fig. 6A), *G. daedala* GdKS1 (Galdieriales) and *Cy. merolae* MS1 (Cyanidioschyzonales; the same order as *Cyanidiococcus* but a different genus) were cultured in Sp medium using a dialysis system at different temperatures. GdKS1 was isolated from Sai-no-Kawara, as was *Cc. yangmingshanensis* CcyaKS1, whereas *Cz. merolae* MS1 (cell-walled diploid) was isolated from Yellowstone National Park (Fig. 6B; Supplementary Fig. 3). *Cyanidioschyzon* and *Cyanidium* were not detected in Kusatsu Hot Spring, and strains previously designated as *Cyanidium* in Japan have been reclassified as *Cyanidiococcus* [62].

Unlike *Cc. yangmingshanensis*, both *Cz. merolae* MS1 and *G. daedala* GdKS1 retained ~40% of their 40°C growth rates at 25°C (Fig. 6C). To examine near-growth-arrest conditions, we compared cells cultured at 40°C with those at 20°C, at which proliferation was minimal, rather than at 25°C (Fig. 6C). Although cell morphology was similar at both temperatures (Fig. 6B and D), protein content per cell was lower at 40°C (Fig. 6E). Absorbance spectra were comparable (Fig. 6F), whereas chlorophyll *a* and phycocyanin contents were lower at 40°C than at 20°C (Fig. 6G), as observed for *Cc. yangmingshanensis.*

**Figure 7:**
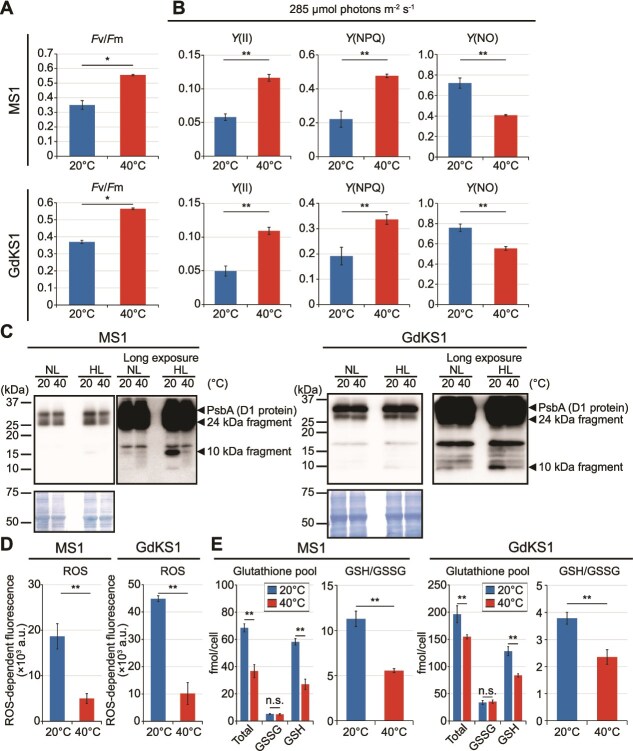
Photosynthetic activities, D1 protein profile, and cellular levels of ROS, GSSG, and GSH in *G. daedala* GdKS1 and *Cz. merolae* MS1 cultured at 40°C and 20°C in Sp medium for 10 days using the dialysis cultivation system. (A) Maximum quantum yield of PSII (*F*v/*F*m) in dark-adapted cells and quantum yield of PSII in light-adapted cells (*F*v′/*F*m′) (B) Effective quantum yield [*Y*(II)], nonphotochemical quenching [*Y*(NPQ)] and nonregulated energy dissipation [*Y*(NO)] measured in light-adapted cells under actinic light of 285 μmol photons m^−2^ s^−1^, approximately equivalent to the cultivation PFD (see Materials and Methods), at 40°C and 20°C. (C) Immunoblot detection of the D1 protein of PSII in cells cultured under 100 μmol photons m^−2^ s^−1^ and after being shifted to 1500 μmol photons m^−2^ s^−1^ for 4 h at 40°C and 20°C. Results of normal and longer exposure times are shown. The same amount (10 μg) of total cellular protein was separated in each lane. A Coomassie Brilliant Blue–stained PVDF membrane is shown as a loading control. (D) Cellular ROS levels quantified using photo-oxidation–resistant DCFH-DA. (E) Cellular concentrations of oxidized glutathione (GSSG), reduced glutathione (GSH), and the GSH/GSSG ratio at 40°C and 20°C. Means ± SDs of four independent cultures are shown for A, B, D, and E (*P < .05; **P < .01; *t-*test).

PAM fluorometry showed that, in both species, *F*v/*F*m were lower at 20°C than at 40°C (Fig. 7A and B; Supplementary Fig. 10), and *Y*(II) (under irradiance close to the culturing light; see Materials and Methods) was similarly reduced (Fig. 7B). At 40°C, excess energy was dissipated through NPQ and *Y*(NO), whereas at 20°C, most excess energy was dissipated via *Y*(NO), as in *Cc. yangmingshanensis* (Fig. 7B; Supplementary Fig. 10).

Immunoblotting showed similar total D1 protein levels at both temperatures (Fig. 7C). However, a 10 kDa D1 degradation fragment accumulated at 20°C, slightly under growth light (100 μmol photons m^−2^ s^−1^) and to a much greater extent under high light (1500 μmol photons m^−2^ s^−1^ for 4 h), whereas only trace levels were detected at 40°C even under high light (Fig. 7C). In addition, intracellular ROS levels, the total glutathione pool, and the GSH/GSSG ratio were higher at 20°C than at 40°C (Fig. 7D and E), similar to those in *Cc. yangmingshanensis.*

## Discussion

### Ecology of Cyanidiophyceae across seasonal temperature gradients in Sai-no-Kawara

At Sai-no-Kawara, Cyanidiophyceae were found at temperatures from 22°C to 47°C; at lower temperatures (<28°C), they consistently coexisted with unicellular green algae and diatoms. Water chemistry remained nearly constant across sites and seasons, whereas temperature varied and positively correlated with Cyanidiophyceae growth (Fig. 1; Supplementary Fig. 1). Such chemically stable conditions throughout the year are also reported for Cyanidiophyceae mats at some sites in Yellowstone National Park (YNP, USA; e.g. Dragon Spring; 46°C–54°C) [63, 64].

Day length at Kusatsu differs by ~1.5-fold between winter and summer solstices (~9.6 vs 14.5 h), and peak irradiance by ~2-fold (~1000 vs. 2000 μmol photons m^−2^ s^−1^). Even in winter, *Cyanidiococcus* proliferated at >26°C, although growth was extremely slow near this lower limit (Fig. 1H; sampled on 4 February 2024). Thus, the seasonal decline in mat coverage at high-flow sites (e.g. Point 1, 33°C) is unlikely due to cell death but rather to detachment, where flow exceeds the reduced growth rate at low temperature. Consistently, *Cyanidiococcus* cells persisted in rock crevices that shielded them from flow at Point 1 after the mat decline (Fig. 1A). Similarly, in Lemonade Creek, spring snowmelt lowers the temperature to ~27°C and increases discharge, resulting in algal mats being swept away [63, 64]. In addition, *Cyanidiococcus* grow year-round above ~26°C in natural habitats, indicating positive net CO₂ fixation; fixed carbon is transferred to unicellular grazers [24] and exported downstream as detached biomass to cooler habitats.

At some sites in YNP, including Lemonade Creek, cyanidiophycean mats also decline during summer, associated with increased irradiance, causing photoinhibition and UV damage [63, 64]. When *Cyanidiococcus* isolated from Sai-no-Kawara was exposed to high light (1500 μmol photons m^−2^ s^−1^), chlorophyll *a*, but not phycocyanin, decreased (Supplementary Fig. 5B), indicating photodamage or downregulation of photosystem cores upon high light stress [4]. However, unlike YNP, no summer mat collapse was observed at Sai-no-Kawara. This contrast may reflect differences in species composition, where *Cyanidiococcus* dominates at Sai-no-Kawara and *Cz. merolae* at YNP, or combined environmental factors such as water chemistry, including toxic heavy metal concentrations.

### Two sides of the gradient: energy dissipation in the cold and nitrogen shortage at the optimum in Cyanidiophyceae

Growth inhibition at low temperature can largely be attributed to reduced enzyme activity. Although 20°C–25°C is not generally considered cold for many algae, Cyanidiophyceae (except secondarily mesophilic genera) are thermophilic, with optimal enzyme temperatures of 42°C–57°C and activities at 25°C reduced to 20%–50% [65–67]. Consistently, both cultured strains and natural populations show maximal growth around 40°C [14, 20, 68, 69] (Fig. 1H). Thus, 20°C–25°C represents a suboptimal low temperature for Cyanidiophyceae.

**Figure 8:**
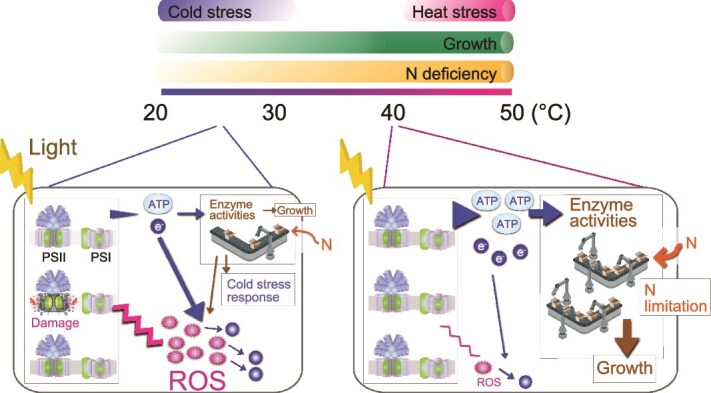
Schematic representation of the physiological states of Cyanidiophyceae across different temperature ranges. (A) Types of stress experienced by cells across different temperature ranges. (B) Comparison of photostasis mechanisms between rapidly growing cells at high temperature and barely proliferating cells at low temperature. At 25°C, where growth is nearly arrested, photosynthetic pigments and apparatus are maintained, leading to continued light absorption despite reduced metabolic demand. Excess excitation energy is largely dissipated through nonregulated pathways, accompanied by an increased fraction of damaged and nonfunctional PSII, leading to elevated ROS production. This excess energy is further consumed by enhanced ROS scavenging via reductive pathways (e.g. GSH, NADPH, ascorbate, and cysteine), as well as by the accumulation of these antioxidants and cold stress responses, including increased mRNA levels and chromosomal maintenance proteins per cell. In contrast, at 40°C, absorbed light energy primarily supports electron transport and biomass production, enabling rapid cellular growth and higher nitrogen demand, which leads to nitrogen limitation responses, with little investment in stress-response pathways. At even higher temperatures, however, cells are expected to incur additional metabolic costs associated with heat shock responses.

At low temperature, cyanidiophycean cells maintained photosynthetic pigments and apparatus components comparable to, or higher than, those of rapidly growing cells, indicating continued light absorption (Figs 1, 3, and 6). Our results suggest that excess energy arising from reduced growth demand, though partly used for homeostasis, is largely dissipated and redirected (Fig. 8): a greater fraction of PSII becomes damaged and nonfunctional, and together with other nonregulated pathways, much absorbed light is lost as fluorescence, heat, or ROS rather than driving electron transport (Figs 4 and 7). The remaining electron flow, reducing power, and ATP are likely redirected from growth to ROS scavenging and detoxification (Figs 5 and 7), as well as to cold-stress responses, including increased chromosomal maintenance and DNA repair proteins, likely reflecting replication stress and elevated repair demand (Supplementary Results; Supplementary Fig. 9). Nucleosomal proteins (Supplementary Results; Supplementary Fig. 9) and total mRNA levels (Supplementary Fig. 6) also increased; the latter likely reflects reduced mRNA turnover rather than enhanced transcription, possibly compensating for delayed rRNA processing and protein synthesis. Although transcription and translation are not expected to be accelerated at low temperature, the increased mRNA pool and chromosomal maintenance proteins per cell imply higher energetic and material costs for building a single cell, thereby increasing consumption of excess NADPH and ATP (cold stress response in Fig. 8). As a result of these responses, a balance between absorbed light energy and its dissipation and use is maintained across temperatures, stabilizing intracellular ATP and redox levels, as observed (Fig. 5D–F; Fig. 8). The decrease in NADP(H) and increase in NAD(H) pools at low temperature observed in this study indicate a shift toward NAD-dependent catabolism, consistent with the distinct roles of NADP in assimilatory and antioxidative reactions and NAD in catabolic processes [21], and with suppressed growth while basal homeostasis is maintained.

In natural habitats, or under laboratory conditions mimicking them that are poor in nitrogen (mostly NH_4_^+^), actively growing cells at 40°C exhibited a nitrogen-deficient state, whereas cells in nutrient-rich media did not. This was evidenced by upregulation of nitrate assimilation genes and reductions in N:C ratio, total protein, and photosynthetic pigments (Fig. 2K), although less pronounced than under complete nitrogen depletion [54, 57, 70]. In addition to the decrease in cellular nitrogen content, the reduction in cellular carbon content (Supplementary Fig. 5A) likely reflects a decline in carbon fixation associated with reduced levels of photosynthetic pigments (Fig. 2K). Although less pronounced than for carbon and nitrogen, the decrease in cellular phosphorus content (Supplementary Fig. 5A) may reflect its relatively sufficient availability relative to cellular demand compared to nitrogen (Fig. 1) while also indicating an overall reduction in biosynthetic activity under these conditions. These responses were weaker at 25°C, suggesting that at 40°C nitrogen supply is insufficient to meet high growth demand. Thus, Cyanidiophyceae face a temperature-dependent trade-off: as temperature decreases below the growth optimum, they increasingly need to dissipate excess energy and cope with oxidative and cold stress, whereas as temperature increases from low values toward the growth optimum, they experience nitrogen deficiency due to limited nitrogen supply relative to elevated metabolic demand.

Within the physiological range, metabolic and growth rates generally increase with temperature due to faster enzymatic reactions. However, chemostat and natural assemblage studies of microalgae, although not direct measurements of in situ growth rates, show that in nutrient-limited environments (e.g. the open ocean), warming-driven growth is constrained because nutrient supply cannot meet increased metabolic demand [71–73]. Our results in Cyanidiophyceae provide a clear example of this phenomenon. In *Cyanidiococcus*, growth at higher temperatures did not further increase in nutrient-rich medium relative to nitrogen-limited spring water (Supplementary Fig. 4B), although nitrogen-deficiency responses disappeared (Fig. 2), suggesting adaptation to persistently low nitrogen availability.

Although not examined in detail here, at even higher temperatures (>42°C), heat stress responses are likely required. Transcriptome analysis of *Cyanidiococcus* in its natural habitat showed that two *HSP20* genes were highly expressed upstream at higher temperatures (44.9°C–45.6°C) but not downstream at lower temperatures (30.0°C–30.5°C) or in dialysis cultures at 25°C–40°C (Supplementary Fig. 11). These genes, acquired by horizontal gene transfer, are induced above 42°C [70], indicating that heat stress responses occur near the upper survival limit. Conversely, at lower temperatures, competition with green algae and diatoms also occurs (Fig. 1E). Thus, Cyanidiophyceae experience stress across their entire temperature range in nature.

The framework proposed in this study (Fig. 8) highlights an ecological strategy in which Cyanidiophyceae, despite having highly reduced gene repertoires, sustain growth under nutrient limitation and tolerate energy imbalance through relatively simple mechanisms, enabling persistence in extreme environments where reduced competition outweighs suboptimal growth.

### Habitats of Cyanidiophyceae and nitrogen availability

Low nitrogen (mainly NH_4_^+^) and relatively high phosphorus (PO_4_^3−^) at Sai-no-Kawara were also observed in three geologically distinct sulfuric hot springs in Japan, each >400 km from Kusatsu where Cyanidiophyceae mats occur (Supplementary Table 11) [14, 24]. Outside Japan, water compositions from sites with Cyanidiophyceae mats have been reported in YNP and in two hot springs in Naples, Italy, with nitrogen concentrations ranging from oligotrophic to extremely high levels depending on site conditions. Although in some cases it is unclear whether samples were collected directly from Cyanidiophyceae mats, nitrogen (mainly NH_4_^+^) varied up to 20-fold over several years but remained within the oligotrophic range (10–50 μM) during low-nitrogen periods (Supplementary Table 11). We further reanalyzed diurnal metatranscriptomic (RNA-seq) data from Lemonade Creek (YNP), focusing on stream mats dominated by *Cz. merolae* (~44°C) and terrestrial mats where *G. yellowstonensis* was also present (~32°C) [74]. Even in these datasets, nitrate-assimilating genes, which are known to be specifically expressed when total nitrogen or NH_4_^+^ was depleted in cultures [54, 57], were more highly expressed at higher temperatures in the stream than at lower temperatures in the soil in *Cz. merolae* (Supplementary Fig. 11). Expression of these genes was also evident in *G. yellowstonensis* in the soil (Supplementary Fig. 11), indicating nitrogen limitation. In contrast, NH_4_^+^ concentration was extremely high in Naples, exceeding 1700 μM (Supplementary Table 11), likely reflecting the characteristics of pool environments. These observations indicate that Cyanidiophyceae habitats are not uniformly nitrogen-poor, yet these algae tolerate and respond to limited nitrogen availability. Such flexibility in nitrogen use may support persistence across spatially and temporally variable environments, particularly when combined with dispersal that enables colonization of newly formed geothermal habitats.

Nitrate was generally much less abundant than NH_4_^+^, although occasional increases were observed (Fig. 1D; Supplementary Fig. 1B; Supplementary Table 11). Although nitrification is often considered limited in acidic environments, it can occur under such conditions [75]. These transient increases likely reflect in situ nitrification under certain conditions, potentially explaining the retention of nitrate assimilation genes in cyanidiophycean lineages.

### Simplified photostasis strategy in Cyanidiophyceae

Across algal and plant lineages, diverse cold-tolerance mechanisms maintain photostasis by balancing light capture with reduced metabolic demand [76]. In many algae and plants, this involves remodeling of the photosynthetic apparatus, including reduced antenna size, enhanced NPQ, and adjustment of the PSI/PSII ratio, together with membrane stabilization via polyunsaturated fatty acids (PUFAs), protein quality control by heat shock proteins (HSPs) and chaperonins, and antioxidant defenses [6, 76–78].

Photosynthetic components in Cyanidiophyceae change little between the lowest habitat temperatures, where growth is minimal, and the optimum, unlike many algae and plants in which substantial remodeling of the photosynthetic apparatus occurs. NPQ decreases at low temperature, and no marked PUFA accumulation occurs (Supplementary Fig. 8). In *C. reinhardtii*, long-term cold treatment altered ~30% of quantified proteins (≥2-fold) [79], whereas in *Cyanidiococcus*, only ~14% changed, mainly proteins involved in oxidative and cold stress responses rather than core metabolism. At the transcript level, only ~2.7% of genes differed ≥2-fold between low and optimal temperatures. This discrepancy between transcriptomic and proteomic responses suggests that temperature adaptation is regulated primarily at post-transcriptional and post-translational levels rather than at the transcriptional level. Thus, the photostasis strategy of Cyanidiophyceae appears simpler than in most phototrophs, consistent with their streamlined genomes (consistency with previous low-temperature studies under nutrient-rich conditions is discussed in Supplementary Discussion). At low temperatures, rather than reducing light absorption, Cyanidiophyceae appear not to downregulate it and rely less on early-stage dissipation of excess excitation energy than many algae. Instead, surplus energy from slow growth is largely diverted to secondary stress responses such as ROS detoxification and cellular maintenance, yet this strategy remains effective under seasonal and spatial temperature variability (Fig. 8).

The simple but effective photostasis strategy (Fig. 8), despite the near-minimal gene sets of Cyanidiophyceae, likely underlies the evolutionary success of Cyanidiophyceae in dynamic geothermal environments. Cyanidiophyceae are not obligate thermophiles and survive for extended periods at lower temperatures (e.g. ~20°C–25°C), although growth is strongly reduced. Cells were found within fragile rock crevices below the water flow (Fig. 1A). Cyanidiophyceae are also known to inhabit endolithic environments [80]; in this study, *Cc. yangmingshanensis* CcyaKSE1 was isolated from an endolithic site (Supplementary Fig. 2). Dispersal by wind or animals has been previously hypothesized [81, 82], and our results support this through tolerance to low temperatures and survival in moist microhabitats within crevices. Together, these observations support persistence across spatially discontinuous geothermal habitats that are continually reshaped.

## Supplementary Material

wrag105_Supplemental_Files

## Data Availability

The RNA- and DNA-sequencing data of *Cc. yangmingshanensis* CcyaKS1 have been deposited in the DDBJ/EMBL/GenBank (BioProject accession no. PRJDB37710; BioSample accession numbers: SAMD01787991–SAMD01788016 and SAMD01789650; DRA run accession numbers: DRR893189–DRR893214 and DRR893215). All the other data generated in this study are included in the manuscript and supplementary data.

## References

[1] Walker RM, Sanabria VC, Youk H. Microbial life in slow and stopped lanes. *Trends Microbiol* 2024;32:650–62. 10.1016/j.tim.2023.11.014 38123400 PMC11187706

[2] Hoehler TM, Jørgensen BB. Microbial life under extreme energy limitation. *Nat Rev Microbiol* 2013;11:83–94. 10.1038/nrmicro2939 23321532

[3] Huner NPA, Ivanov AG, Wilson KE., et al. Energy Sensing and Photostasis in Photoautotrophs. In: Storey KB, Storey JM (eds). Cell and Molecular Response to Stress. Amsterdam: Elsevier, 2002, 243–55 10.1016/S1568-1254(02)80019-5.

[4] Nishiyama Y, Murata N. Revised scheme for the mechanism of photoinhibition and its application to enhance the abiotic stress tolerance of the photosynthetic machinery. *Appl Microbiol Biotechnol* 2014;98:8777–96. 10.1007/s00253-014-6020-0 25139449

[5] Vass I . Molecular mechanisms of photodamage in the Photosystem II complex. *Biochim Biophys Acta* 2012;1817:209–17. 10.1016/j.bbabio.2011.04.014 21565163

[6] Ermilova E . Cold stress response: an overview in *Chlamydomonas*. *Front Plant Sci* 2020;11:569437. 10.3389/fpls.2020.569437 33013991 PMC7494811

[7] Hüner NPA, Bode R, Dahal K., et al. Shedding some light on cold acclimation, cold adaptation, and phenotypic plasticity. *Botany* 2013;91:127–36. 10.1139/cjb-2012-0174

[8] Niyogi KK, Truong TB. Evolution of flexible nonphotochemical quenching mechanisms that regulate light harvesting in oxygenic photosynthesis. *Curr Opin Plant Biol* 2013;16:307–14. 10.1016/j.pbi.2013.03.011 23583332

[9] Bellaflore S, Barneche F, Peltler G., et al. State transitions and light adaptation require chloroplast thylakoid protein kinase STN7. *Nature* 2005;433:892–5. 10.1038/nature03286 15729347

[10] Fujimori T, Hihara Y, Sonoike K. PsaK2 subunit in photosystem I is involved in state transition under high light condition in the cyanobacterium *Synechocystis* sp. PCC 6803. *J Biol Chem* 2005;280:22191–7. 10.1074/jbc.M500369200 15824118

[11] Hihara Y, Sonoike K. Regulation, Inhibition and Protection of Photosystem I. In: Aro EM, Andersson B (eds). Regulation of Photosynthesis. Netherlands: Springer, 2001, 507–31 10.1007/0-306-48148-0_29.

[12] Cho CH, Park SI, Huang TY., et al. Genome-wide signatures of adaptation to extreme environments in red algae. *Nat Commun* 2023;14:10. 10.1038/s41467-022-35566-x 36599855 PMC9812998

[13] Miyagishima S, Tanaka K. The unicellular red alga *Cyanidioschyzon merolae* - The simplest model of a photosynthetic eukaryote. *Plant Cell Physiol* 2021;62:926–41. 10.1093/pcp/pcab052 33836072 PMC8504449

[14] Hirooka S, Fujiwara T, Seger M., et al. Sexual life cycle establishes the unicellular red algae Cyanidiophyceae as a genetically tractable model lineage for eukaryotic evolution. *bioRxiv* 2025. 10.1101/2025.10.15.681507 PMC13353863 42246676

[15] Qiu H, Price DC, Weber APM., et al. Adaptation through horizontal gene transfer in the cryptoendolithic red alga *Galdieria phlegrea*. *Curr Biol* 2013;23:R865–6. 10.1016/j.cub.2013.08.046 24112977

[16] Van Etten J, Cho CH, Yoon HS., et al. Extremophilic red algae as models for understanding adaptation to hostile environments and the evolution of eukaryotic life on the early earth. *Semin Cell Dev Biol* 2023;134:4–13. 10.1016/j.semcdb.2022.03.007 35339358

[17] Kumazawa M, Ifuku K. Unraveling the evolutionary trajectory of LHCI in red-lineage algae: Conservation, diversification, and neolocalization. *iScience* 2024;27:110897. 10.1016/j.isci.2024.110897 39386759 PMC11462038

[18] Lee JM, Kim D, Bhattacharya D., et al. Expansion of phycobilisome linker gene families in mesophilic red algae. *Nat Commun* 2019;10:4823. 10.1038/s41467-019-12779-1 31645564 PMC6811547

[19] Doemel WN, Brock TD. The physiological ecology of *Cyanidium caldarium*. *J Gen Microbiol* 1971;67:17–32. 10.1099/00221287-67-1-17

[20] Brock TD . Thermophilic microorganisms and life at high temperatures. New York, NY: Springer New York, 1978, 10.1007/978-1-4612-6284-8.

[21] Lu Z, Ren T, Li Y., et al. Nutrient limitations on photosynthesis: from individual to combinational stresses. *Trends Plant Sci* 2025;30:872–85. 10.1016/j.tplants.2025.03.006 40221269

[22] Saroussi S, Sanz-Luque E, Kim RG., et al. Nutrient scavenging and energy management: acclimation responses in nitrogen and sulfur deprived *Chlamydomonas*. *Curr Opin Plant Biol* 2017;39:114–22. 10.1016/j.pbi.2017.06.002 28692856

[23] Van Mooy BAS, Fredricks HF, Pedler BE., et al. Phytoplankton in the ocean use nonphosphorus lipids in response to phosphorus scarcity. *Nature* 2009;458:69–72. 10.1038/nature07659 19182781

[24] Sunada Y, Tsujino D, Yamashita S., et al. Heterotrophic unicellular eukaryotes feeding on the unicellular red alga *Cyanidiococcus* sp. in moderately hot geothermal sulfuric springs. *FEMS Microbiol Ecol* 2025;101:fiaf048. 10.1093/femsec/fiaf048 40307847 PMC12063673

[25] Ohnuma M, Yokoyama T, Inouye T., et al. Polyethylene glycol (PEG)-mediated transient gene expression in a red alga, *Cyanidioschyzon merolae* 10D. *Plant Cell Physiol* 2008;49:117–20. 10.1093/pcp/pcm157 18003671

[26] Edler D, Klein J, Antonelli A., et al. raxmlGUI 2.0: A graphical interface and toolkit for phylogenetic analyses using RAxML. *Methods Ecol Evol* 2021;12:373–7. 10.1111/2041-210X.13512

[27] Kozlov AM, Darriba D, Flouri T., et al. RAxML-NG: A fast, scalable and user-friendly tool for maximum likelihood phylogenetic inference. *Bioinformatics* 2019;35:4453–5. 10.1093/bioinformatics/btz305 31070718 PMC6821337

[28] Allen MB . Studies with *Cyanidium caldarium*, an anomalously pigmented chlorophyte. *Arch Microbiol* 1959;32:270–7. 10.1007/BF00409348 13628094

[29] Jong LW, Fujiwara T, Hirooka S., et al. Cell size for commitment to cell division and number of successive cell divisions in cyanidialean red algae. *Protoplasma* 2021;258:1103–18. 10.1007/s00709-021-01628-y 33675395

[30] Yamashita S, Hirooka S, Fujiwara T., et al. Costs of photosynthesis and cellular remodeling in trophic transitions of the unicellular red alga *Galdieria partita*. *Commun Biol* 2025;8:891. 10.1038/s42003-025-08284-5 40483364 PMC12145455

[31] Hirooka S, Itabashi T, Ichinose TM., et al. Life cycle and functional genomics of the unicellular red alga *Galdieria* for elucidating algal and plant evolution and industrial use. *Proc Natl Acad Sci USA* 2022;119:e2210665119. 10.1073/pnas.2210665119 36194630 PMC9565259

[32] Arnon DI, McSwain BD, Tsujimoto HY., et al. Photochemical activity and components of membrane preparations from blue-green algae I. Coexistence of two photosystems in relation to chlorophyll *a* and removal of phycocyanin. *Biochim Biophys Acta* 1974;357:231–45. 10.1016/0005-2728(74)90063-2 4153919

[33] Wellburn AR . The spectral determination of chlorophylls *a* and *b*, as well as total carotenoids, using various solvents with spectrophotometers of different resolution. *J Plant Physiol* 1994;144:307–13. 10.1016/S0176-1617(11)81192-2

[34] Ota S, Yoshihara M, Yamazaki T., et al. Deciphering the relationship among phosphate dynamics, electron-dense body and lipid accumulation in the green alga *Parachlorella kessleri*. *Sci Rep* 2016;6:srep25731. 10.1038/srep25731 PMC4867602 27180903

[35] Ota S, Kawano S. Extraction and molybdenum blue-based quantification of total phosphate and polyphosphate in *Parachlorella*. *Bio Protoc* 2017;7:e2539. 10.21769/bioprotoc.2539 PMC8413496 34541192

[36] Bradford MM . A rapid and sensitive method for the quantitation of microgram quantities of protein utilizing the principle of protein-dye binding. *Anal Biochem* 1976;72:248–54. 10.1016/0003-2697(76)90527-3 942051

[37] Oxborough K, Baker NR. Resolving chlorophyll *a* fluorescence images of photosynthetic efficiency into photochemical and nonphotochemical components – calculation of *qP* and *Fv*′/*Fm*′ without measuring *Fo*′. *Photosynth Res* 1997;54:135–42. 10.1023/A:1005936823310

[38] Kramer DM, Johnson G, Kiirats O., et al. New fluorescence parameters for the determination of QA redox state and excitation energy fluxes. *Photosynth Res* 2004;79:209–18. 10.1023/B:PRES.0000015391.99477.0d 16228395

[39] Kodru S, A u R, Vass I. Chloramphenicol enhances photosystem II photodamage in intact cells of the cyanobacterium *Synechocystis* PCC 6803. *Photosynth Res* 2020;145:227–35. 10.1007/s11120-020-00784-1 32979144 PMC7541379

[40] Nishiyama Y, Yamamoto H, Allakhverdiev SI., et al. Oxidative stress inhibits the repair of photodamage to the photosynthetic machinery. *EMBO J* 2001;20:5587–94. 10.1093/emboj/20.20.5587 11598002 PMC125664

[41] Minoda A, Sakagami R, Yagisawa F., et al. Improvement of culture conditions and evidence for nuclear transformation by homologous recombination in a red alga, *Cyanidioschyzon merolae* 10D. *Plant Cell Physiol* 2004;45:667–71. 10.1093/pcp/pch087 15215501

[42] Li H . Minimap2: Pairwise alignment for nucleotide sequences. *Bioinformatics* 2018;34:3094–100. 10.1093/bioinformatics/bty191 29750242 PMC6137996

[43] Koren S, Walenz BP, Berlin K., et al. Canu: Scalable and accurate long-read assembly via adaptive k-mer weighting and repeat separation. *Genome Res* 2017;27:722–36. 10.1101/gr.215087.116 28298431 PMC5411767

[44] Camacho C, Coulouris G, Avagyan V., et al. BLAST+: Architecture and applications. *BMC Bioinformatics* 2009;10:421. 10.1186/1471-2105-10-421 20003500 PMC2803857

[45] Kim D, Paggi JM, Park C., et al. Graph-based genome alignment and genotyping with HISAT2 and HISAT-genotype. *Nat Biotechnol* 2019;37:907–15. 10.1038/s41587-019-0201-4 31375807 PMC7605509

[46] Park SI, Cho CH, Ciniglia C., et al. Revised classification of the Cyanidiophyceae based on plastid genome data with descriptions of the Cavernulicolales ord. nov. and Galdieriales ord. nov. (Rhodophyta). *J Phycol* 2023;59:444–66. 10.1111/jpy.13322 36792488

[47] Cho CH, Park SI, Ciniglia C., et al. Potential causes and consequences of rapid mitochondrial genome evolution in thermoacidophilic *Galdieria* (Rhodophyta). *BMC Evol Biol* 2020;20:112. 10.1186/s12862-020-01677-6 32892741 PMC7487498

[48] Bolger AM, Lohse M, Usadel B. Trimmomatic: A flexible trimmer for Illumina sequence data. *Bioinformatics* 2014;30:2114–20. 10.1093/bioinformatics/btu170 24695404 PMC4103590

[49] Love MI, Huber W, Anders S. Moderated estimation of fold change and dispersion for RNA-seq data with DESeq2. *Genome Biol* 2014;15:550. 10.1186/s13059-014-0550-8 25516281 PMC4302049

[50] Kabeya Y, Miyagishima S. Chloroplast DNA replication is regulated by the redox state independently of chloroplast division in *Chlamydomonas reinhardtii*. *Plant Physiol* 2013;161:2102–12. 10.1104/pp.113.216291 23447524 PMC3613479

[51] Dodds WK, Smith VH. Nitrogen, phosphorus, and eutrophication in streams. *Inland Waters* 2016;6:155–64. 10.5268/IW-6.2.909

[52] Gross W . Ecophysiology of algae living in highly acidic environments. *Hydrobiol* 2000;433:31–7. 10.1023/A:1004054317446

[53] Ferris MJ, Sheehan KB, Kühl M., et al. Algal species and light microenvironment in a low-pH, geothermal microbial mat community. *Appl Environ Microbiol* 2005;71:7164–71. 10.1128/AEM.71.11.7164-7171.2005 16269755 PMC1287733

[54] Imamura S, Kanesaki Y, Ohnuma M., et al. R2R3-type MYB transcription factor, CmMYB1, is a central nitrogen assimilation regulator in *Cyanidioschyzon merolae*. *Proc Natl Acad Sci USA* 2009;106:12548–53. 10.1073/pnas.0902790106 19592510 PMC2718362

[55] Sekine K, Sakakibara Y, Hase T., et al. A novel variant of ferredoxin-dependent sulfite reductase having preferred substrate specificity for nitrite in the unicellular red alga *Cyanidioschyzon merolae*. *Biochem J* 2009;423:91–8. 10.1042/BJ20090581 19622064

[56] Sanz-Luque E, Chamizo-Ampudia A, Llamas A., et al. Understanding nitrate assimilation and its regulation in microalgae. *Front Plant Sci* 2015;6:899. 10.3389/fpls.2015.00899 26579149 PMC4620153

[57] Fujiwara T, Kanesaki Y, Hirooka S., et al. A nitrogen source-dependent inducible and repressible gene expression system in the red alga *Cyanidioschyzon merolae*. *Front Plant Sci* 2015;6:657. 10.3389/fpls.2015.00657 26379685 PMC4549557

[58] Sumiya N, Kawase Y, Hayakawa J., et al. Expression of cyanobacterial acyl-ACP reductase elevates the triacylglycerol level in the red alga *Cyanidioschyzon merolae*. *Plant Cell Physiol* 2015;56:1962–80. 10.1093/pcp/pcv120 26272551

[59] Fujiwara T, Hirooka S, Ohbayashi R., et al. Relationship between cell cycle and diel transcriptomic changes in metabolism in a unicellular red alga. *Plant Physiol* 2020;183:1484–501. 10.1104/pp.20.00469 32518202 PMC7401142

[60] Telfer A . Singlet oxygen production by PSII under light stress: Mechanism, detection and the protective role of β-carotene. *Plant Cell Physiol* 2014;55:1216–23. 10.1093/pcp/pcu040 24566536 PMC4080269

[61] Haubetauhl K . A chloroplast DegP2 protease performs the primary cleavage of the photodamaged D1 protein in plant photosystem II. *EMBO J* 2001;20:713–22. 10.1093/emboj/20.4.713 11179216 PMC145409

[62] Liu S, Chiang Y, Yoon HS., et al. Comparative genome analysis reveals *Cyanidiococcus* gen. nov., a new extremophilic red algal genus sister to *Cyanidioschyzon* (Cyanidioschyzonaceae, Rhodophyta). *J Phycol* 2020;56:1428–42. 10.1111/jpy.13056 33460076

[63] Lehr CR, Frank SD, Norris TB., et al. Cyanidia (cyanidiales) population diversity and dynamics in an acid-sulfate-chloride spring in Yellowstone National Park. *J Phycol* 2007;43:3–14. 10.1111/j.1529-8817.2006.00293.x

[64] Skorupa DJ, Reeb V, Castenholz RW., et al. Cyanidiales diversity in Yellowstone National Park. *Lett Appl Microbiol* 2013;57:459–66. 10.1111/lam.12135 23865641

[65] Ford TW . Ribulose 1,5-bisphosphate carboxylase from the thermophilic, acidophilic alga, *Cyanidium caldarium* (Geitler). *Biochim Biophys Acta* 1979;569:239–48. 10.1016/0005-2744(79)90059-7 113034

[66] Murakami M, Osanai T. Biochemical properties of β-amylase from red algae and improvement of its thermostability through immobilization. *ACS Omega* 2022;7:36195–205. 10.1021/acsomega.2c03315 36278071 PMC9583313

[67] Yamamoto M, Osanai T, Ito S. l-Lactate dehydrogenase from *Cyanidioschyzon merolae* shows high catalytic efficiency for pyruvate reduction and is inhibited by ATP. *Plant Mol Biol* 2024;114:98. 10.1007/s11103-024-01495-0 39254882 PMC11387445

[68] Rigano C, Violante U. Comparative growth of the thermal alga *Cyanidium caldarium* on nitrate and ammonia at different temperatures. *Arch Microbiol* 1972;85:13–8. 10.1007/BF00425139 5072724

[69] Rossoni AW, Schönknecht G, Lee HJ., et al. Cold acclimation of the thermoacidophilic red alga *Galdieria sulphuraria*: Changes in gene expression and involvement of horizontally acquired genes. *Plant Cell Physiol* 2019;60:702–12. 10.1093/pcp/pcy240 30590832

[70] Sumiya N, Fujiwara T, Kobayashi Y., et al. Development of a heat-shock inducible gene expression system in the red alga *Cyanidioschyzon merolae*. *PLoS One* 2014;9:e111261. 10.1371/journal.pone.0111261 25337786 PMC4206486

[71] Marañón E, Lorenzo MP, Cermeño P., et al. Nutrient limitation suppresses the temperature dependence of phytoplankton metabolic rates. *ISME J* 2018;12:1836–45. 10.1038/s41396-018-0105-1 29695860 PMC6018665

[72] Fernández-González C, Tarran GA, Schuback N., et al. Phytoplankton responses to changing temperature and nutrient availability are consistent across the tropical and subtropical Atlantic. *Commun Biol* 2022;5:1035. 10.1038/s42003-022-03971-z 36175608 PMC9522883

[73] Liu K, Suzuki K, Chen B., et al. Are temperature sensitivities of *Prochlorococcus* and *Synechococcus* impacted by nutrient availability in the subtropical northwest Pacific? *Limnol Oceanogr* 2021;66:639–51. 10.1002/lno.11629

[74] Stephens TG, Van Etten J, McDermott T., et al. Temporal dynamics in a red alga dominated geothermal feature in Yellowstone National Park. *ISME Commun* 2024;4:ycae151. 10.1093/ismeco/ycae151 39711979 PMC11662350

[75] Ni G, Leung PM, Daebeler A., et al. Nitrification in acidic and alkaline environments. *Essays Biochem* 2023;67:753–68. 10.1042/EBC20220194 37449414 PMC10427799

[76] Hüner NPA, Ivanov AG, Szyszka-Mroz B., et al. Photostasis and photosynthetic adaptation to polar life. *Photosynth Res* 2024;161:51–64. 10.1007/s11120-024-01104-7 38865029

[77] Satyakam , Zinta G, Singh RK., et al. Cold adaptation strategies in plants—An emerging role of epigenetics and antifreeze proteins to engineer cold resilient plants. *Front Genet* 2022;13:909007. 10.3389/fgene.2022.909007 36092945 PMC9459425

[78] Chang T, Zhao Y, He H., et al. Exogenous melatonin improves growth in hulless barley seedlings under cold stress by influencing the expression rhythms of circadian clock genes. *PeerJ* 2021;9:e10740. 10.7717/peerj.10740 33552735 PMC7831369

[79] Valledor L, Furuhashi T, Hanak A-M., et al. Systemic cold stress adaptation of *Chlamydomonas reinhardtii*. *Mol Cell Proteomics* 2013;12:2032–47. 10.1074/mcp.M112.026765 23564937 PMC3734567

[80] Gross W, Tischendorf G, Küver J., et al. Cryptoendolithic growth of the red alga *Galdieria sulphuraria* in volcanic areas. *Eur J Phycol* 1998;33:25–31. 10.1080/09670269810001736503

[81] Ciniglia C, Yang EC, Pollio A., et al. Cyanidiophyceae in Iceland: plastid *rbc*L gene elucidates origin and dispersal of extremophilic *Galdieria sulphuraria* and *G. maxima* (Galdieriaceae, Rhodophyta). *Phycologia* 2014;53:542–51. 10.2216/14-032.1

[82] Toplin JA, Norris TB, Lehr CR., et al. Biogeographic and phylogenetic diversity of thermoacidophilic cyanidiales in Yellowstone National Park, Japan, and New Zealand. *Appl Environ Microbiol* 2008;74:2822–33. 10.1128/AEM.02741-07 18344337 PMC2394875

