# Supplementary material for: Temperature-dependent photostasis and nitrogen limitation in streamlined-genome red algae Cyanidiophyceae from natural habitats
Source: ISME J. 2026 Apr 30;20(1):wrag105. doi: 10.1093/ismejo/wrag105 (PMC13200283; doi:10.1093/ismejo/wrag105)
Supplement: wrag105_Supplemental_Files [file wrag105_supplemental_files.zip › Supplymentary_Fig_F_wrag105.pdf]

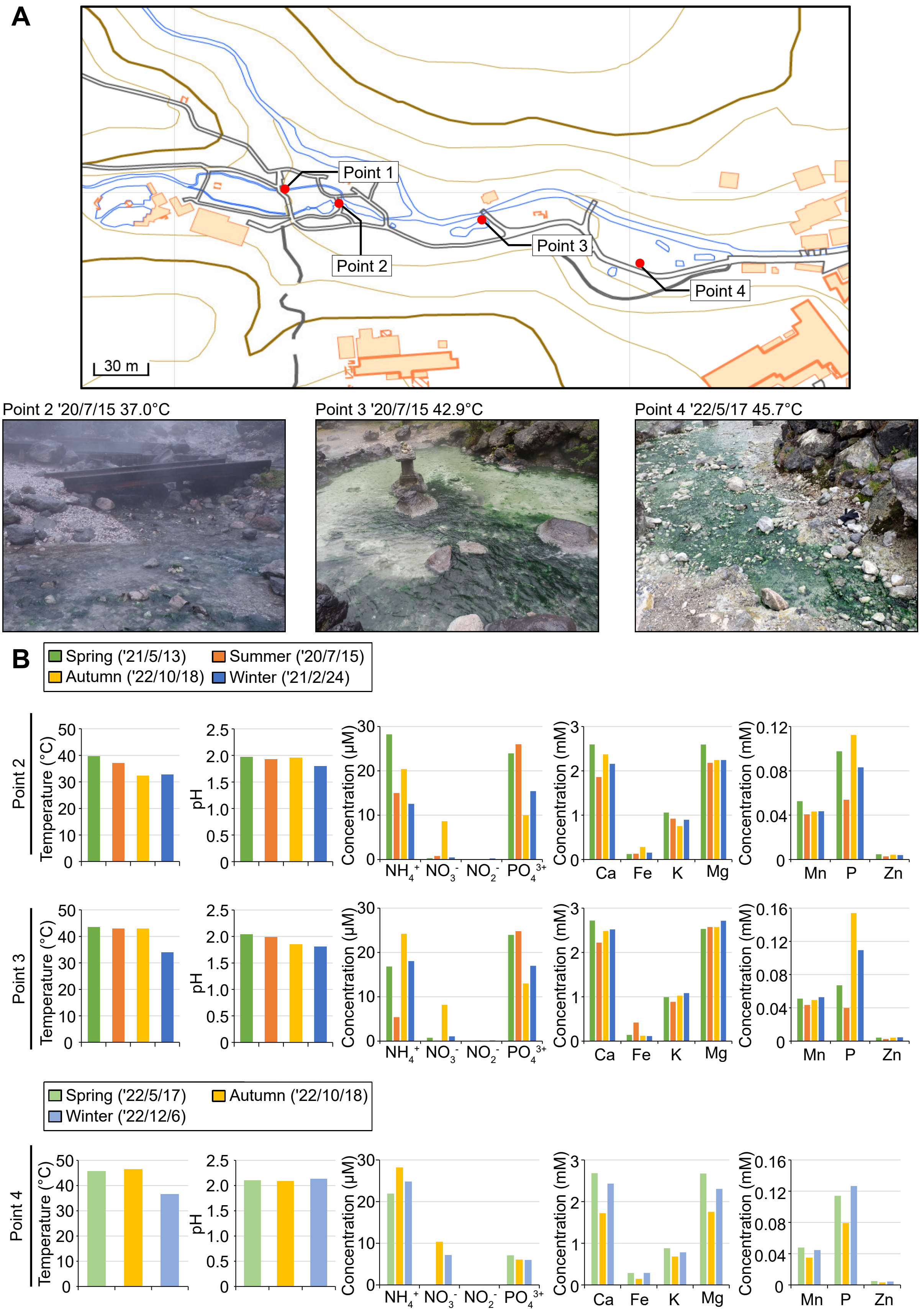
A
Point 1
Point 2
Point 3
Point 4
30 m
Point 2 '20/7/15 37.0°C
Point 3 '20/7/15 42.9°C
Point 4 '22/5/17 45.7°C
B
Spring ('21/5/13)
Summer ('20/7/15)
Autumn ('22/10/18)
Winter ('21/2/24)
Point 2
Point 3
Temperature (°C)
pH
Concentration (µM)
Concentration (mM)
NH4+ NO3- NO2- PO43+
Ca Fe K Mg
Mn P Zn
Spring ('22/5/17)
Autumn ('22/10/18)
Winter ('22/12/6)
Point 4

Fig.S1

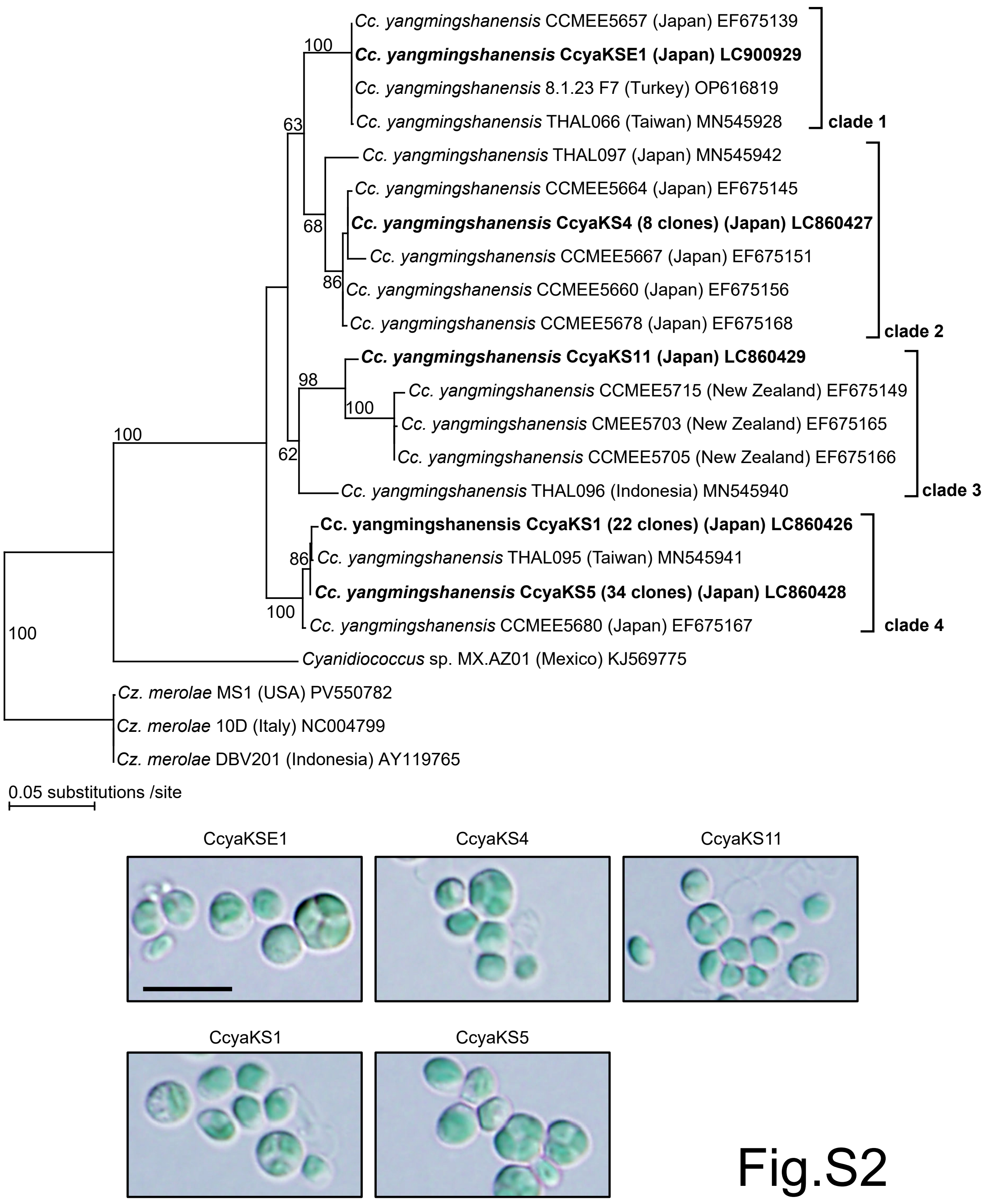
Cc. yangmingshanensis CCMEE5657 (Japan) EF675139
Cc. yangmingshanensis CcyaKSE1 (Japan) LC900929
Cc. yangmingshanensis 8.1.23 F7 (Turkey) OP616819
Cc. yangmingshanensis THAL066 (Taiwan) MN545928
clade 1
Cc. yangmingshanensis THAL097 (Japan) MN545942
Cc. yangmingshanensis CCMEE5664 (Japan) EF675145
Cc. yangmingshanensis CcyaKS4 (8 clones) (Japan) LC860427
Cc. yangmingshanensis CCMEE5667 (Japan) EF675151
Cc. yangmingshanensis CCMEE5660 (Japan) EF675156
Cc. yangmingshanensis CCMEE5678 (Japan) EF675168
clade 2
Cc. yangmingshanensis CcyaKS11 (Japan) LC860429
Cc. yangmingshanensis CCMEE5715 (New Zealand) EF675149
Cc. yangmingshanensis CMEE5703 (New Zealand) EF675165
Cc. yangmingshanensis CCMEE5705 (New Zealand) EF675166
Cc. yangmingshanensis THAL096 (Indonesia) MN545940
clade 3
Cc. yangmingshanensis CcyaKS1 (22 clones) (Japan) LC860426
Cc. yangmingshanensis THAL095 (Taiwan) MN545941
Cc. yangmingshanensis CcyaKS5 (34 clones) (Japan) LC860428
Cc. yangmingshanensis CCMEE5680 (Japan) EF675167
clade 4
Cyanidiococcus sp. MX.AZ01 (Mexico) KJ569775
Cz. merolae MS1 (USA) PV550782
Cz. merolae 10D (Italy) NC004799
Cz. merolae DBV201 (Indonesia) AY119765
100
63
68
86
98
100
62
100
86
100
100
0.05 substitutions /site
CcyaKSE1
CcyaKS4
CcyaKS11
CcyaKS1
CcyaKS5

Fig.S2

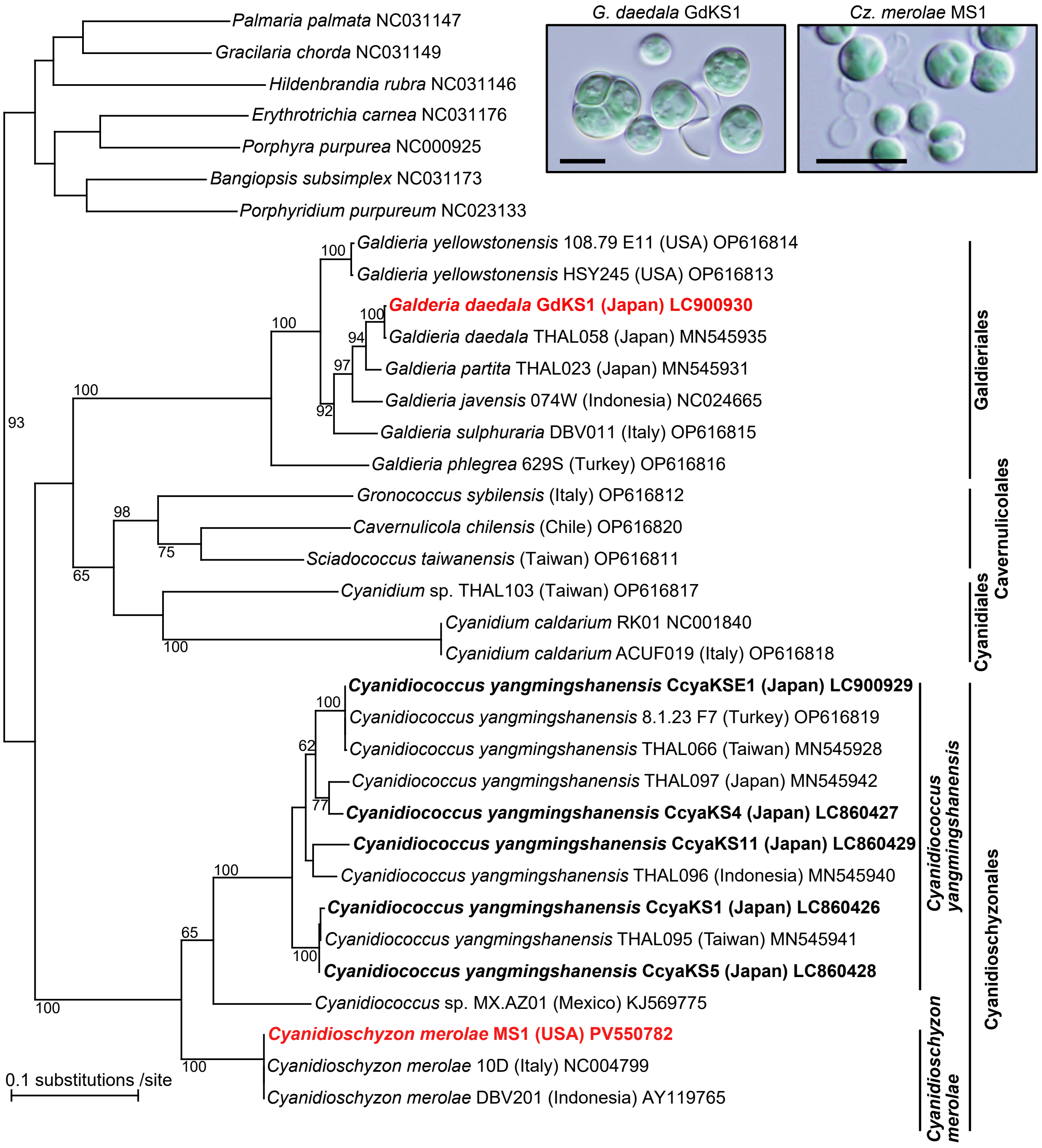
G. daedala GdKS1
Cz. merolae MS1
Palmaria palmata NC031147
Gracilaria chorda NC031149
Hildenbrandia rubra NC031146
Erythrotrichia carnea NC031176
Porphyra purpurea NC000925
Bangiopsis subsimplex NC031173
Porphyridium purpureum NC023133
Galdieria yellowstonensis 108.79 E11 (USA) OP616814
Galdieria yellowstonensis HSY245 (USA) OP616813
Galderia daedala GdKS1 (Japan) LC900930
Galdieria daedala THAL058 (Japan) MN545935
Galdieria partita THAL023 (Japan) MN545931
Galdieria javensis 074W (Indonesia) NC024665
Galdieria sulphuraria DBV011 (Italy) OP616815
Galdieria phlegrea 629S (Turkey) OP616816
Gronococcus sybilensis (Italy) OP616812
Cavernulicola chilensis (Chile) OP616820
Sciadococcus taiwanensis (Taiwan) OP616811
Cyanidium sp. THAL103 (Taiwan) OP616817
Cyanidium caldarium RK01 NC001840
Cyanidium caldarium ACUF019 (Italy) OP616818
Cyanidiococcus yangmingshanensis CcyaKSE1 (Japan) LC900929
Cyanidiococcus yangmingshanensis 8.1.23 F7 (Turkey) OP616819
Cyanidiococcus yangmingshanensis THAL066 (Taiwan) MN545928
Cyanidiococcus yangmingshanensis THAL097 (Japan) MN545942
Cyanidiococcus yangmingshanensis CcyaKS4 (Japan) LC860427
Cyanidiococcus yangmingshanensis CcyaKS11 (Japan) LC860429
Cyanidiococcus yangmingshanensis THAL096 (Indonesia) MN545940
Cyanidiococcus yangmingshanensis CcyaKS1 (Japan) LC860426
Cyanidiococcus yangmingshanensis THAL095 (Taiwan) MN545941
Cyanidiococcus yangmingshanensis CcyaKS5 (Japan) LC860428
Cyanidiococcus sp. MX.AZ01 (Mexico) KJ569775
Cyanidioschyzon merolae MS1 (USA) PV550782
Cyanidioschyzon merolae 10D (Italy) NC004799
Cyanidioschyzon merolae DBV201 (Indonesia) AY119765
0.1 substitutions /site
93
100
100
100
100
94
97
92
98
75
65
100
100
62
77
100
65
100
100
100
Galdieriales
Cavernulicolales
Cyanidiales
Cyanidiococcus yangmingshanensis
Cyanidioschyzon merolae
Cyanidioschyzonales

Fig.S3

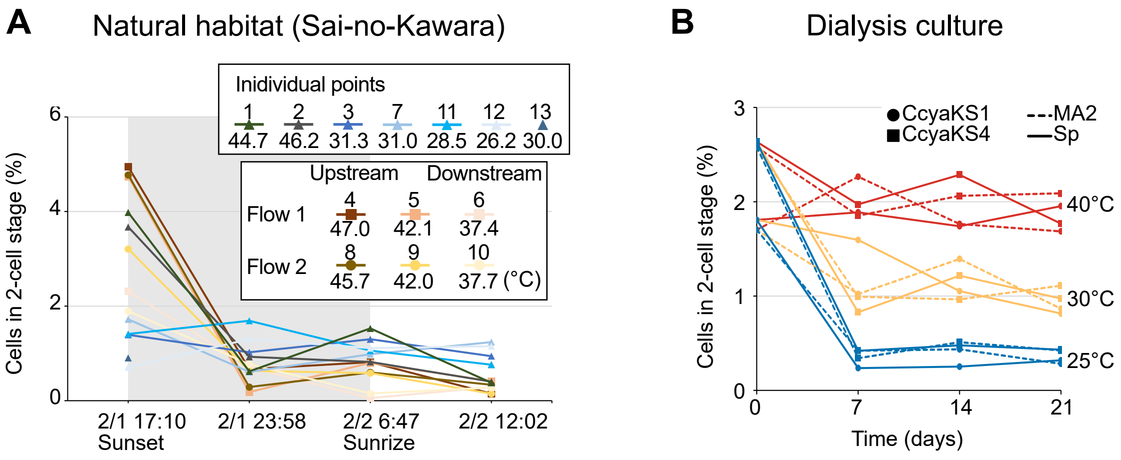
A
Natural habitat (Sai-no-Kawara)
Inidividual points
1 44.7
2 46.2
3 31.3
7 31.0
11 28.5
12 26.2
13 30.0
Upstream
Downstream
Flow 1
4 47.0
5 42.1
6 37.4
Flow 2
8 45.7
9 42.0
10 37.7 (°C)
Cells in 2-cell stage (%)
0
2
4
6
2/1 17:10
Sunset
2/1 23:58
2/2 6:47
Sunrize
2/2 12:02
B
Dialysis culture
●CcyaKS1
■CcyaKS4
---MA2
—Sp
40°C
30°C
25°C
Cells in 2-cell stage (%)
0
1
2
3
0
7
14
21
Time (days)

Fig.S4

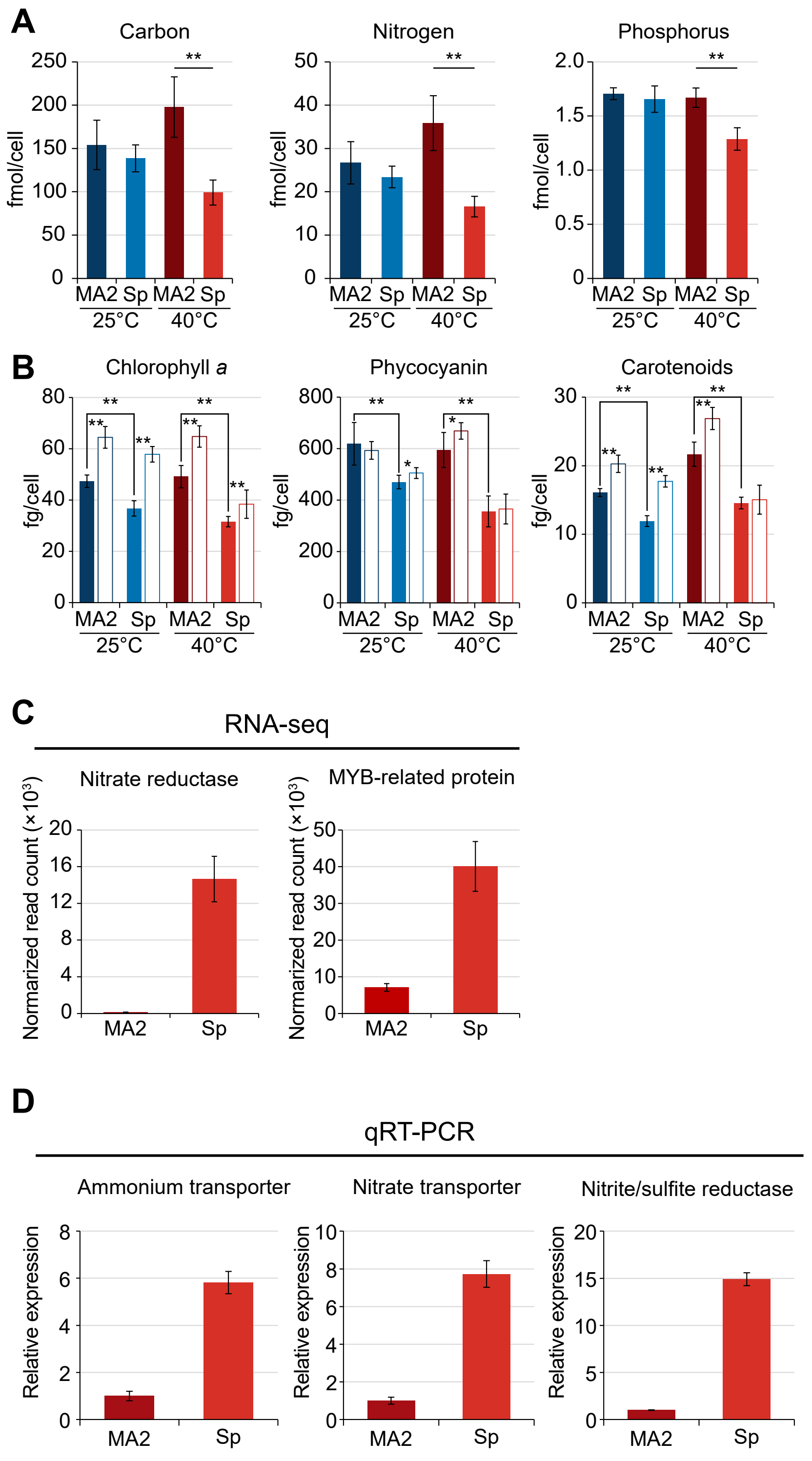
A
Carbon
Nitrogen
Phosphorus
fmol/cell
MA2 Sp MA2 Sp
25°C 40°C
B
Chlorophyll a
Phycocyanin
Carotenoids
fg/cell
C
RNA-seq
Nitrate reductase
MYB-related protein
Normarized read count (×10³)
D
qRT-PCR
Ammonium transporter
Nitrate transporter
Nitrite/sulfite reductase
Relative expression

Fig.S5

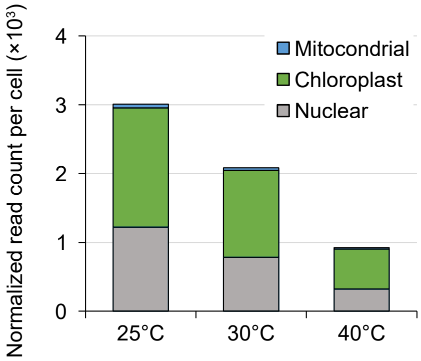

Normalized read count per cell (×10³)
4
3
2
1
0
Mitocondrial
Chloroplast
Nuclear
25°C
30°C
40°C

Fig.S6

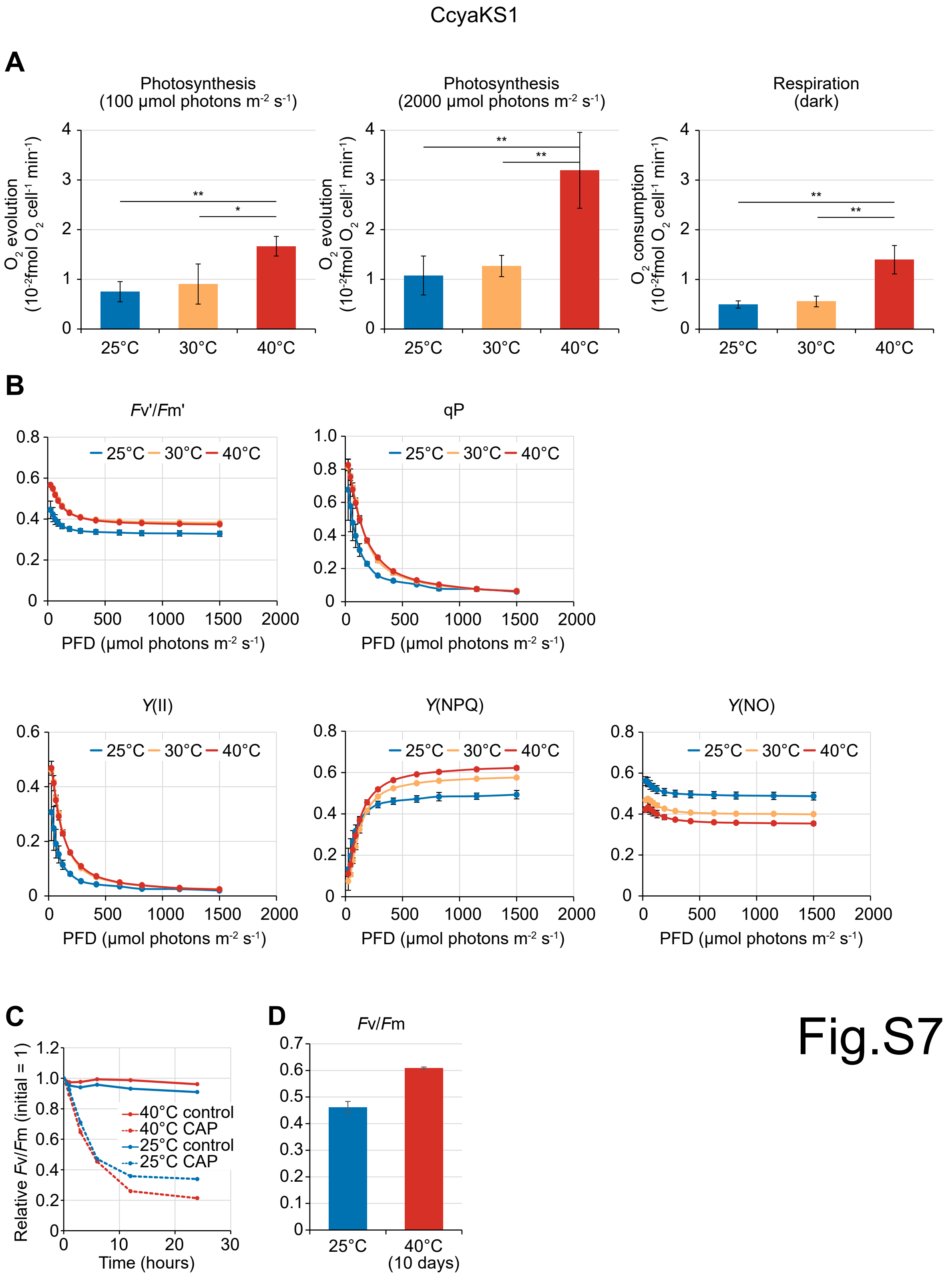

CcyaKS1
A
Photosynthesis
(100 μmol photons m-2 s-1)
Photosynthesis
(2000 μmol photons m-2 s-1)
Respiration
(dark)
O2 evolution
(10-2fmol O2 cell-1 min-1)
O2 consumption
(10-2fmol O2 cell-1 min-1)
25°C
30°C
40°C
B
Fv'/Fm'
qP
Y(II)
Y(NPQ)
Y(NO)
PFD (μmol photons m-2 s-1)
C
Relative Fv/Fm (initial = 1)
Time (hours)
40°C control
40°C CAP
25°C control
25°C CAP
D
Fv/Fm
25°C
40°C
(10 days)

Fig.S7

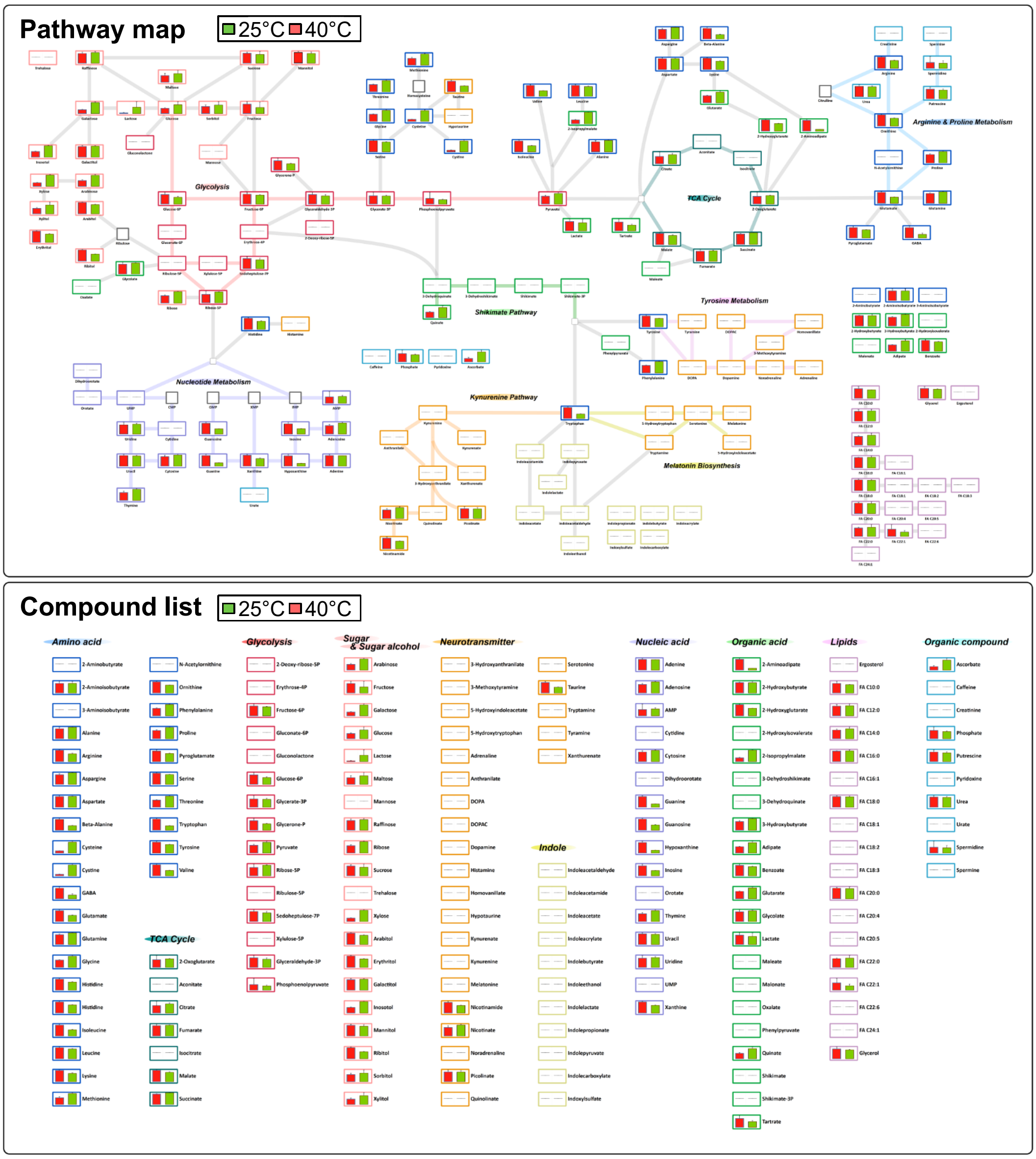
Pathway map
25°C 40°C
Glycolysis
TCA Cycle
Arginine & Proline Metabolism
Shikimate Pathway
Tyrosine Metabolism
Nucleotide Metabolism
Kynurenine Pathway
Melatonin Biosynthesis
Compound list
25°C 40°C
Amino acid
Glycolysis
Sugar & Sugar alcohol
Neurotransmitter
Nucleic acid
Organic acid
Lipids
Organic compound
TCA Cycle
Indole

Fig.S8

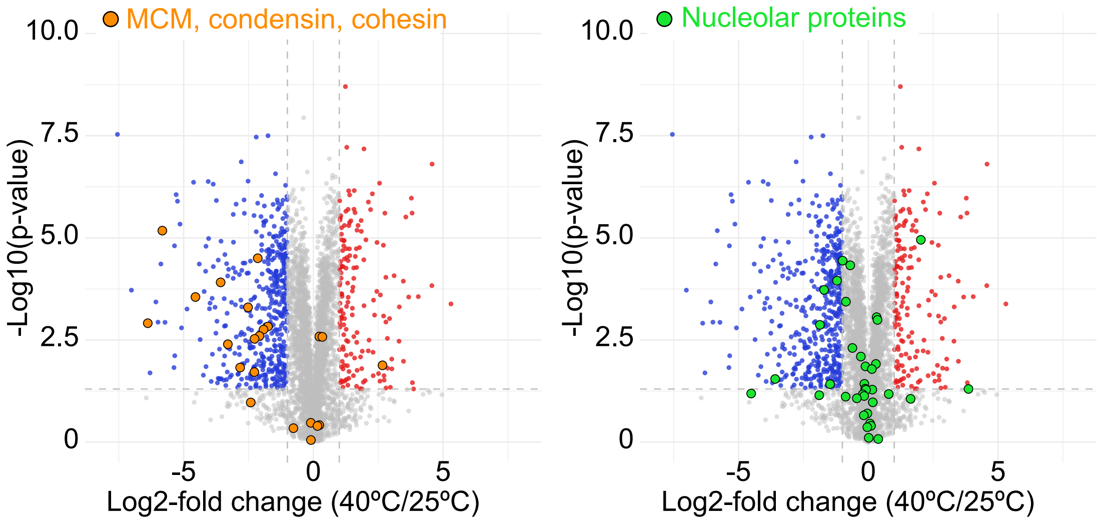
MCM, condensin, cohesin
10.0
7.5
5.0
2.5
0
-Log10(p-value)
-5
0
5
Log2-fold change (40ºC/25ºC)
Nucleolar proteins
10.0
7.5
5.0
2.5
0
-Log10(p-value)
-5
0
5
Log2-fold change (40ºC/25ºC)

Fig.S9

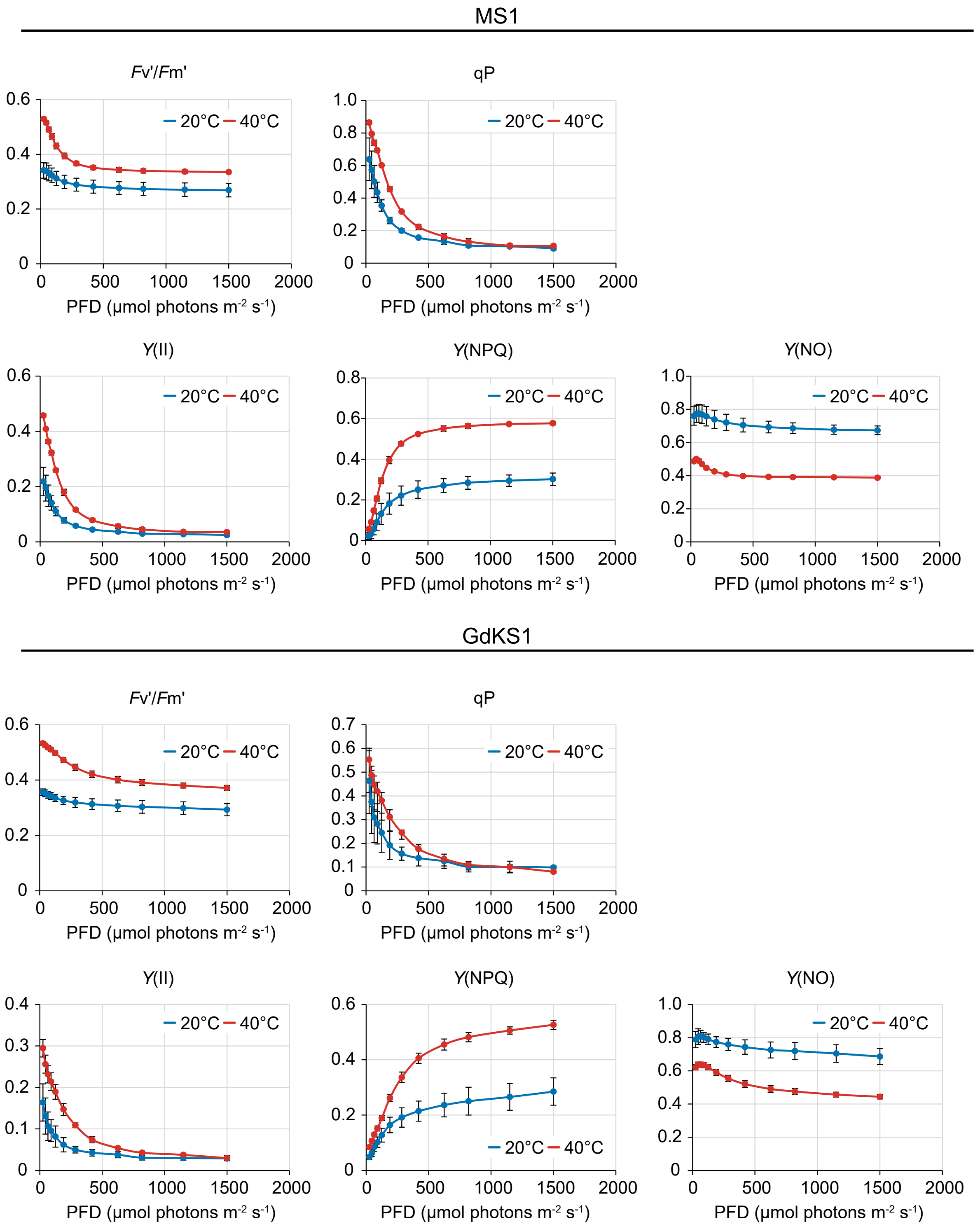

Fig.S10

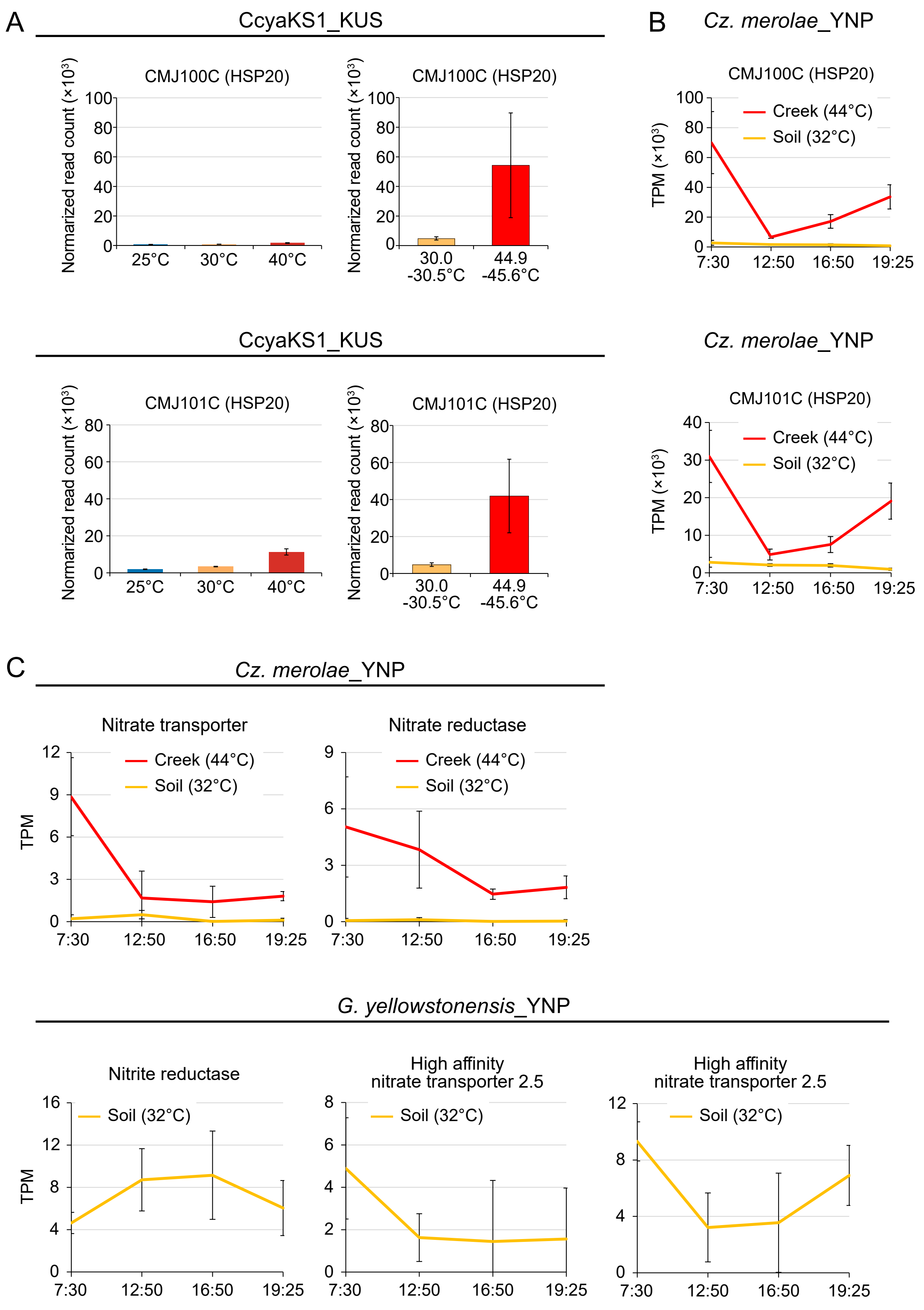
A
CcyaKS1_KUS
CMJ100C (HSP20)
Normarized read count (×10³)
100
80
60
40
20
0
25°C
30°C
40°C
30.0
-30.5°C
44.9
-45.6°C
CMJ101C (HSP20)
B
Cz. merolae_YNP
TPM (×10³)
Creek (44°C)
Soil (32°C)
7:30
12:50
16:50
19:25
C
Nitrate transporter
Nitrate reductase
TPM
G. yellowstonensis_YNP
Nitrite reductase
High affinity
nitrate transporter 2.5

Fig.S11

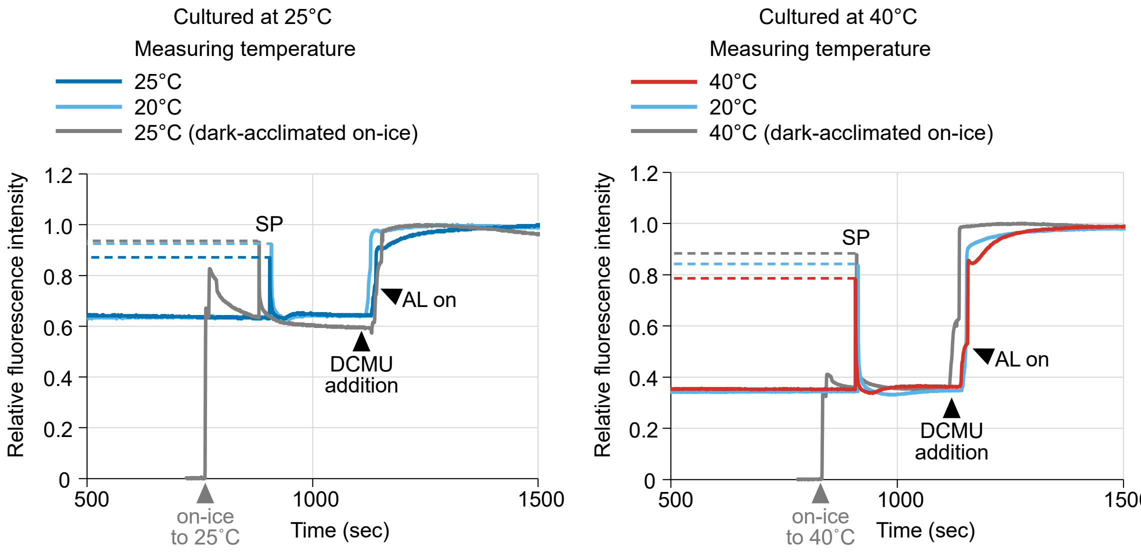
Cultured at 25°C
Measuring temperature
25°C
20°C
25°C (dark-acclimated on-ice)
Relative fluorescence intensity
1.2
1.0
0.8
0.6
0.4
0.2
0
SP
AL on
DCMU addition
500
1000
1500
on-ice to 25°C
Time (sec)
Cultured at 40°C
Measuring temperature
40°C
20°C
40°C (dark-acclimated on-ice)
Relative fluorescence intensity
SP
AL on
DCMU addition
on-ice to 40°C
Time (sec)

Fig.S12
